# Multi-Variate EEG Analysis as a Novel Tool to Examine Brain Responses to Naturalistic Music Stimuli

**DOI:** 10.1371/journal.pone.0141281

**Published:** 2015-10-28

**Authors:** Irene Sturm, Sven Dähne, Benjamin Blankertz, Gabriel Curio

**Affiliations:** 1 Berlin School of Mind and Brain, Humboldt Universität zu Berlin, Berlin, Germany; 2 Neurotechnology Group, Technische Universität Berlin, Berlin, Germany; 3 Neurophysics Group, Department of Neurology, Charité University Medicine, Berlin, Germany; 4 Bernstein Focus Neurotechnology, Berlin, Germany; 5 Bernstein Center for Computational Neuroscience, Berlin, Germany; University Zurich, SWITZERLAND

## Abstract

Note onsets in music are acoustic landmarks providing auditory cues that underlie the perception of more complex phenomena such as beat, rhythm, and meter. For naturalistic ongoing sounds a detailed view on the neural representation of onset structure is hard to obtain, since, typically, stimulus-related EEG signatures are derived by averaging a high number of identical stimulus presentations. Here, we propose a novel multivariate regression-based method extracting onset-related brain responses from the ongoing EEG. We analyse EEG recordings of nine subjects who passively listened to stimuli from various sound categories encompassing simple tone sequences, full-length romantic piano pieces and natural (non-music) soundscapes. The regression approach reduces the 61-channel EEG to one time course optimally reflecting note onsets. The neural signatures derived by this procedure indeed resemble canonical onset-related ERPs, such as the N1-P2 complex. This EEG projection was then utilized to determine the Cortico-Acoustic Correlation (CACor), a measure of synchronization between EEG signal and stimulus. We demonstrate that a significant CACor (i) can be detected in an individual listener's EEG of a single presentation of a full-length complex naturalistic music stimulus, and (ii) it co-varies with the stimuli’s average magnitudes of sharpness, spectral centroid, and rhythmic complexity. In particular, the subset of stimuli eliciting a strong CACor also produces strongly coordinated tension ratings obtained from an independent listener group in a separate behavioral experiment. Thus musical features that lead to a marked physiological reflection of tone onsets also contribute to perceived tension in music.

## Introduction

Music organizes sound in time [1]. Since single sound events with a distinct onset are known to elicit evoked EEG responses in the brain, such as the P1-N1-P2 complex [2], music can be hypothesized to organize also our brain activity in time: a temporal sequence of musical tone onsets can be assumed to be echoed by a sequence of event-related potentials (ERPs) in the brain signal.

Onset-related ERP responses have been studied in numerous contexts in the music domain [3], [4], [5] and in the speech domain [6] The common approach to examine cortical onset responses is to present a large number of identical stimuli and average across these presentations, thereby enhancing the signal-to-noise ratio in order to make ERPs visible. Obviously, this puts constraints on complexity as well as duration of the material that can be presented. Moreover, music listening is a process that strongly depends on situational variables, such as the listener’s state and listening strategy. Consequently, brain responses will also be influenced by variables that change from presentation to presentation. This aspect has been addressed explicitly recently in Jäncke et al. [7] and can be regarded as strong argument for a single-presentation analysis in the context of music listening.

Thus, if averaging is not feasible, e.g., when full-length naturalistic pieces of music are presented, alternative ways must be established to extract stimulus-related activity that is hidden in a mixture of brain activity unrelated to the stimulus and signals from non-cerebral sources from the ongoing EEG.

In the domain of speech processing cortical onset responses that reflect changes in the waveform envelope (termed Envelope Following Responses, EFRs), have been a target of interest for a long time [8], [9], [10]. Continued research on topics such as speech comprehension promoted a methodological progress from analyzing EFRs to short distinct sounds towards those of naturalistic speech. Several approaches aiming at extracting EFRs from continuous EEG or MEG have been proposed that, e.g., combine source reconstruction techniques with explicit modeling of the N1-P2 complex with convolution models [11] or with spatial filtering methods [12] or estimate the impulse response of the auditory system [13], [14]. These methods have provided a distinct picture of the brain signals ‘following’ the speech waveform envelope, but still rely on a relatively high number of stimulus repetitions.

In the domain of (natural) music processing EEG-reflections of the sound envelope for single presentations of a full-length complex piece of music have (to our knowledge) been only addressed once before: In [7] the relationship between band-power modulations in several frequency bands in the theta, alpha and beta range and the stimulus envelope has been examined. The band-power modulations varied across a large number of repetitions of the same stimulus consistently in a large number of subjects, and, thus, were taken to reflect rather dynamic aspects of listening to music, but not invariant, stimulus-dependent aspects. More general attempts have been made to track the stimulus structure in the continuous (broadband) EEG. In a classification approach Schaefer et al. [15] decoded to which of seven 3s-fragments of original music participants were listening to from their EEG and extracted a N1-like EEG component related to sound envelope changes. Cong et al. [16] and Thompson et al. [17] applied ICA-related techniques and extracted EEG components predicting the envelope of the stimulus waveform that were detected reliably in a set of EEG recordings of either a group of subjects that listens to the same piece of music [16] or of one subject who listens to a variety of musical excerpts [17]. These findings were obtained in an unsupervised manner, i.e., without prior assumptions about musical aspects, and delineate a general relationship between EEG and music stimulus that can be detected at subject-group level and across a range of music stimuli.

Given these results and the susceptibility of the onset-related N1-P2 response to a variety of stimulus-related, subject-related and situational influences, it is an interesting question whether envelope-following responses to musical stimuli can be utilized to obtain valuable information about aspects of music perception. Insights into this question could be obtained by exploring the relationship between EEG signal and stimulus envelope in detail, examining influences of acoustic factors and subject variables, and, importantly, assessing the behavioral/experiential relevance of this phenomenon. However, an approach dedicated to examining the relationship between EEG and waveform envelope in a stimulus-specific and subject-individual manner has not been established to our knowledge. Yet, such a method would be instrumental for expanding the scope from properties of single tones (e.g., timbre of acoustic stimuli experienced during practice [18], [19]) to complex musical properties that unfold over time, such as rhythmic regularity or other complex patterns. Beyond that, such a technical advance would allow to address aspects that imply naturalistic stimulation, such as cultural references (style, genre), listener preferences, or familiarity in the music domain.

Here, we propose a novel approach that aims to extract brain responses to tone onsets in continuous music from the EEG. We utilize the fact that the rapid changes in sound intensity which indicate note onsets in music are captured by the first derivative of the audio waveform’s envelope. Exchanging ‘envelope’, the feature on which existing Envelope-Following-Response techniques are based (see above), with the equivalent feature ‘power’, we denote the key audio feature in the present analysis ‘audio power slope’ in the following. We apply Ridge Regression with temporal embedding to train spatio-temporal filters that optimize the correlation between the 61-channel EEG of single subjects and the power slope of the audio signal. This yields a one-dimensional projection of the EEG that can subsequently be examined at various time scales. We analyse EEG recordings of subjects (N = 9) who listened to nine stimuli from different sound categories and examine the resulting Cortico-Acoustic Correlation (CACor), the correlation between a regression-derived EEG projection and an audio power slope at the level of a single subject and a single presentation. We relate global stimulus descriptions derived by acoustic waveform analysis to CACor scores for the nine stimuli in order to learn how stimulus characteristics influence the presence of significant CACor in single presentations for different stimuli.

In the present context, we wish to complement a possible link between stimulus structure and brain signal with a measure of subjective experience at various time scales. Examples from the visual domain suggest that, in principle, such links may exist [20]. Here, as a first step towards creating a connection to an emotion-related dimension of music listening, we employ the concept of musical tension. Musical tension refers to the continuous build-up and subsiding of excitement that can be experienced when listening to music [21]. As a fast-changing component of emotion with a clear relation to musical structure [22], tension has become a well-established concept in music psychology (see [23] for an overview). In particular, it has been attributed as being ‘perceptual objective’ [24] due to a high reliability for repeated measurements [25] and ‘immanent in the music itself’ [26]. Therefore, we preferred the concept of tension to dimensions of more general models of emotion, such as the valence-arousal model [27] where an influence of stimulus-unrelated influences might be stronger.

In order to explore the relation between CACor and behaviorally reported measures of music experience we compare CACor scores to coordination measures derived from continuous subjective tension ratings recorded in a separate behavioral experiment. Finally, we investigate the relation between time-resolved group-level CACor, tension ratings, and the dynamics of musical parameters within each stimulus in order to obtain a more detailed view on the determinants that influence the reflection of onset structure in the EEG and/or lead to consistent reports of tension/relaxation in a group of listeners.

## Materials and Methods

**Fig 1:**
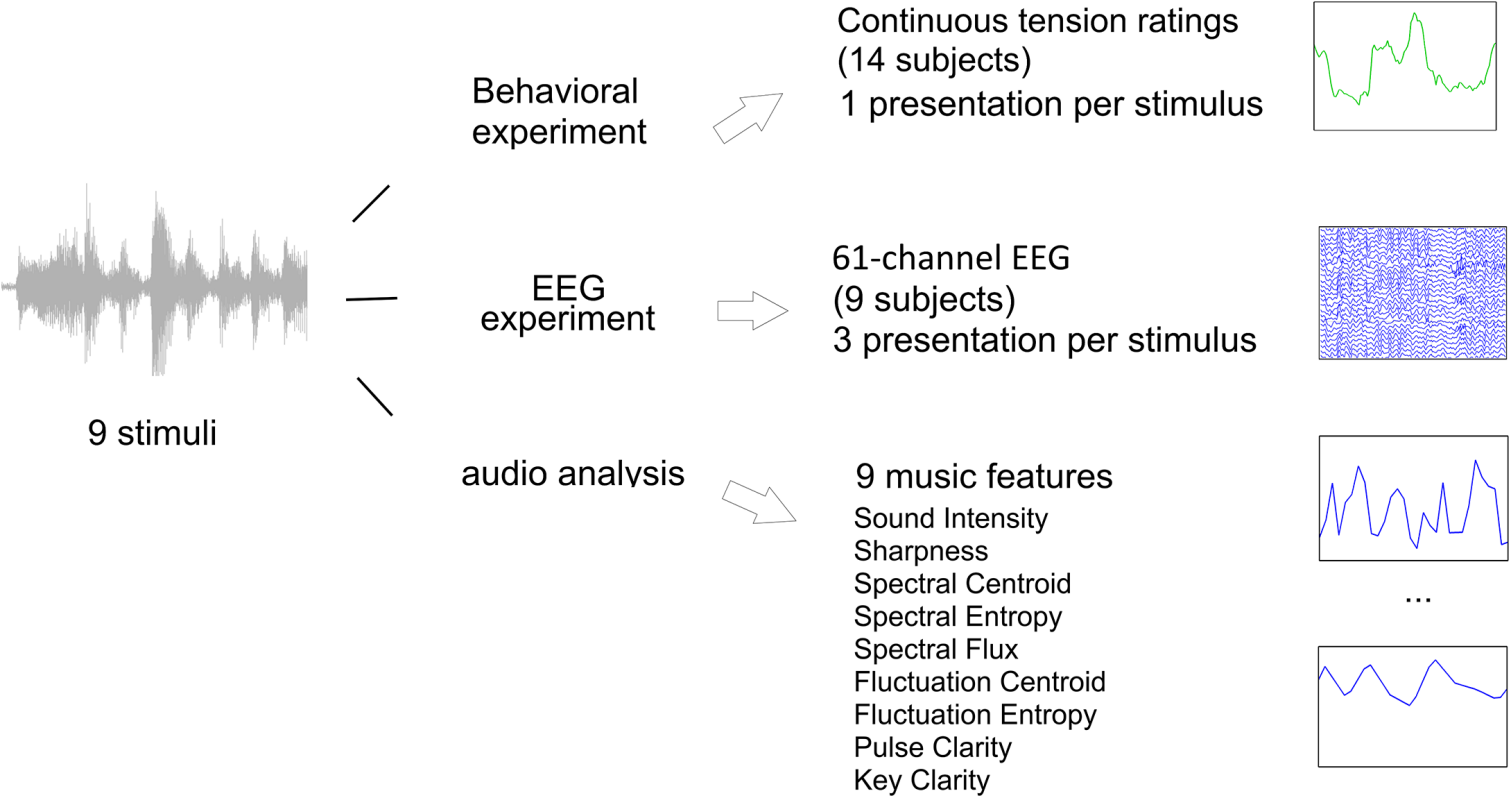
Overview of experiments and data types. In the present study data related to the processing of a set of music and non-music sounds is obtained in three steps. In a behavioral experiment 14 participants gave continuous tension ratings while listening. This resulted in one time course of tension ratings for each stimulus and subject. In an EEG experiment the brain signals of nine subjects were recorded while they listened to the stimuli. Stimuli were repeated three times for each stimulus, resulting in 27 EEG recordings for each stimulus. From the waveform of each stimulus a set of nine acoustic/musical features was extracted from each stimulus.

In the present study data related to a set of nine music and non-music sound clips is obtained in three steps that are summarized in Fig 1. First, in a behavioral experiment 14 participants gave continuous ratings of perceived musical tension while listening to eight of the nine sound clips; second in an EEG experiment the brain signals of nine subjects were recorded while they listened three times to all nine stimuli. Finally, a set of nine acoustic/musical features was extracted from the waveform of each of the nine stimuli. The details for each of these procedures are given below.

### Participants

#### Participants EEG experiment

Nine participants (6 male, 3 female), aged 24–44 years (mean age 30), all but one right-handed, volunteered to take part in the experiment. All reported having normal hearing and no history of neurological disorder. The subjects differed with respect to their musical education and practice: two of them reported intensive musical training of more than 15 years and on more than one instrument, five of them modest amounts of musical training (mean: 7 years) and two of them no musical training beyond obligatory lessons at school. Subjects completed a questionnaire about their musical activities and preferences. Participants gave written consent and the study was performed in accordance with the Declaration of Helsinki. The study protocol was approved by the Ethics Committee of the Charité University Medicine Berlin.

#### Participants behavioral experiment

In a separate experiment 14 new participants (9 male, 5 female, 12 right-handed, 2 left-handed) volunteered to take part in the behavioral experiment. Their musical experience ranged from no musical training beyond obligatory courses at school to more than 15 years of musical training (mean 6 years).

### Apparatus

Brain activity was recorded with multi-channel EEG amplifiers (BrainAmp hardware, BrainProducts, Germany) using 63 Ag/AgCl electrodes (mounted on a Fast’n’Easy cap, Easycap, Germany) in an extended 10–20 system sampled at 5000 Hz with a band-pass from 0.05 to 200 Hz. All skin-electrode impedances were kept below 20 k. Additionally, horizontal and vertical electrooculograms (EOG) were recorded. Signals of scalp electrodes were referenced against a nose electrode. Music stimuli were presented in mono format using Sennheiser PMX 200 Neckband Closed Back Headphones. The stimuli were normalized, so that the peak volumes of each sound file attained the same level. Prior to the experiment, the loudness was adjusted to a level that was convenient for the individual participant using a short excerpt of Vivaldi, Spring (which was representative for the dynamic range of all stimuli).This setting was not changed during the experiment, so that differences in loudness between the stimuli were comparable between subjects. The audio signal was recorded as an additional EEG channel for accurate synchronization.

In the behavioral experiment tension ratings were recorded using a custom-made joystick that was operated with the thumb of the dominant hand. A small spring integrated into the joystick allowed to indicate the build-up of tension by pushing the joystick upwards and decreasing tension by releasing the joystick. The joystick position was sampled at 50 Hz.

### Stimuli

Nine stimuli from different sound categories were presented in the experiment (see S1 Table for more detailed information): (1) Badinerie by J.S. Bach, (2) The Four Seasons, Spring, by A. Vivaldi, (3) The Four Seasons, Summer, by A. Vivaldi, (4) Etude op. 12, No. 10, by F. Chopin, (5) Prelude op. 32, No. 5, by S. Rachmaninov, (6) Theme of Schindler’s List, by J. Williams, (7) an isochronous sequence of major triad chords with root notes on all tones of the chromatic scale (chord duration 350 ms, including rise and fall times of 17.5 ms, interonset interval (IOI) 420 ms, after 7–11 repetitions of a chord change to a new chord in random manner), (8) Jungle noise and (9) instrumental noise.

### Procedure

#### EEG experiment

The present study aims at approximating listening situations that resemble those of everyday life. Consequently, participants were not given any special task and were just asked to listen in a relaxed manner and with closed eyes during the presentation of the musical stimuli.

The main experiment was split into three blocks. In each block the nine stimuli were presented in a different order that was designed such that two piano pieces, two violin pieces or two non-musical stimuli never occurred in direct succession.

#### Behavioral experiment

In the behavioral experiment subjects were given a short introduction to the concept of tension in music. Then, they were instructed as following:

“In the following you listen to eight excerpts, divided by short breaks. Six of them are musical pieces, two are non-musical. Your task is to indicate continuously with the joystick how you experience the evolution of tension of each piece of music. Please start each piece with the joystick at zero position. If you experience an increase in tension in the music, move the joystick up. If you experience a decrease in tension, release the joystick towards the zero position. You will have the opportunity to practice this before the beginning of the experiment. These tension ratings reflect your individual experience. Therefore it is not possible to do this right or wrong.”

After one practice trial with a different music stimulus, each music stimulus was presented once while joystick movements were recorded. Since the stimulus Chord sequence, due to its simplicity and repetitiveness, was not assumed to give rise to the perception of tension in the listeners it was not part of the behavioral experiment.

### Data Analysis

#### Audio analysis

For each stimulus the power slope was determined by segmenting the audio signal into 50% overlapping time frames of 50 ms width and then calculating the average power of each window. Subsequently, the resulting time course was smoothed using a Gaussian filter of three samples width and the first derivative was taken. Then, the extracted power slope was resampled to match the sampling frequency of the EEG.

We chose a set of nine musical features that cover a broad spectrum of timbral, tonal, and rhythmic categories of music. Sound intensity, which can be considered as an approximate measure of loudness, is a stimulus feature that influences a variety of brain responses [28], [2], [29]. Sharpness, defined as the mean positive first derivative of the waveform power [30], has been found to be an important cue for cortical tracking of the speech envelope [31], [30]. Furthermore, with Spectral centroid, Spectral entropy and Spectral flux we included a set of three spectral features that describe pitch- and timbre-related aspects of sounds. The fluctuation spectrum of an audio signal contains the periodicities contained in a sound wave’s envelope. For musical sounds with rhythmic regularity peaks in the fluctuation spectrum correspond to beat-related frequencies. The fluctuation spectrum can be further characterized by the Fluctuation centroid that indicates where the ‘center of mass’ of the spectrum is and by Fluctuation entropy which is a measure of rhythmic complexity. Pulse clarity is a composite feature that indicates how easily listeners perceive the underlying rhythmic or metrical pulsation of a piece of music. It has been introduced and perceptually validated in [32] and since then has been used in numerous studies [33], [34], [35], [36], [37], [38]. Key clarity is a feature that estimates the salience of key.

Sound intensity and Sharpness were calculated in Matlab (The MathWorks Inc., Natick, Massachusetts). All other features were extracted using the MIRToolbox [39]. Sound intensity and the three spectral features were calculated for time frames of 50 ms overlapping by 50%. Sharpness, Fluctuation centroid and Fluctuation entropy, Pulse clarity and Key clarity were determined for time frames of 3s with a 33% overlap. Subsequently, all features were re-sampled to match the sampling rate of the time-resolved CACor of 3.3 Hz. Additionally, for each stimulus a global description was obtained by taking the mean of each music feature in order to derive a rough estimation of the specific characteristics of each stimulus.

#### Preprocessing of tension ratings

In a first step the 14 continuous tension ratings for each stimulus were examined with respect to their homogeneity within the group. This was done by calculating all pairwise correlation coefficients between the single subjects’ ratings. Next, the percentage of significantly correlated pairs was determined with a permutation testing approach where surrogate versions of all 14 tension ratings were generated (see Section Methods, Subsection ‘Significance of correlation‘). With percentages of significantly correlated pairs of tension ratings ranging between 32% and 38% for the non-musical stimuli and Williams and between 47% and 76% for the other music stimuli calculating mean tension ratings for further analysis seemed appropriate. When examining the relationship between tension ratings and stimulus parameters it has to be considered that typically ratings lag behind the stimulus. We utilize the influence of sound intensity on tension ratings that has been reported in the literature [22], [40], [21] to determine the optimal time lag between tension ratings and stimulus. We calculate the cross-correlation between the Grand Average z-score-transformed tension ratings and sound intensity for time lags from 0 to 3s in steps of 10 ms and identify an optimal lag for each stimulus. The resulting time lags ranged from 760 to 1110 ms for the music stimuli, for the non-musical stimuli cross-correlation sequences did not reveal a clear peak. Therefore we set the time lag to 1000 ms. All results related to tension ratings in the following were obtained after correcting for these time lags and re-sampling to the sampling rate of the time-resolved CACor of 3.33 Hz.

In addition to group averages, collections of tension ratings can also be examined with respect to the degree of inter-subject coordination a stimulus exerts on the ratings. Following the framework of activity analysis [41] we determined for each of the 50%-overlapping 1000 ms time frames the percentage of increasing tension ratings (out of the 14 ratings of all subjects) and that of decreasing ratings. Subsequently, we evaluated whether these proportions are significant by applying a permutation-based testing framework (for details see [42]). This resulted in a set of time windows with significantly coordinated rating activity for each stimulus. This can be used to determine a global Coordination Score for each stimulus that indicates how much significantly coordinated rating activity (either increasing or decreasing) occurs in the course of the stimulus. This measure allows comparing stimuli with respect to their ‘ability’ to coordinate behavioral responses.

#### EEG analysis: preprocessing and ERP analysis

Prior to the regression analysis generic preprocessing was performed on the EEG data. The EEG data was lowpass-filtered using a Chebyshev filter (with passbands and stopbands of 42 Hz and 49 Hz, respectively) and downsampled to 100 Hz. After that, a 1-Hz highpass-filter was applied. The EEG signal we used for further analysis, therefore, is a broadband signal in the range of 1 to 42 Hz. In principle, one could assume that the EEG features of interest in this analysis may be contained in the low frequency bands, e.g., in the theta and alpha band. Our preliminary analyses, however, showed that the best match between EEG projection and audio power slope can be obtained using the broadband signal.

Since electrodes A1 and A2 were not contained in the head model used in the later analysis (see Section ‘EEG analysis: Calculating Cortico-Acoustic Correlation‘) they were not considered in the analysis. In order to remove signal components of non-neural origin, such as eye, muscle or movement artifacts while preserving the overall temporal structure of the music-related EEG responses we separated the 61-channel EEG data into independent components using the TDSEP algorithm (Temporal Decorrelation source SEParation, [43], with time lags of tau = 0, …990 ms). ICA components that were considered as purely or predominantly driven by artifacts based on visual inspection of power spectrum, time course and topography (see also [44] and [45]; the Supplementary Material of [44] contains example plots of typical components of various types of artifacts.) were discarded and the remaining components were projected back into the original sensor space.

The stimulus Chord Sequence is an isochronous sequence of chords that only differ in frequency content. This represented an opportunity to apply a conventional ERP analysis approach for comparison with the results of the proposed method. The continuous EEG data was segmented into epochs of 300 ms length, starting at tone onsets, and averaged. Since the pre-processing procedure included already highpass-filtering, a baseline correction was not applied. This technique cannot be applied to any other of the stimuli, owing to the high variability of onset characteristics in naturalistic sounds.

#### EEG analysis: Calculating Cortico-Acoustic Correlation

**Fig 2:**
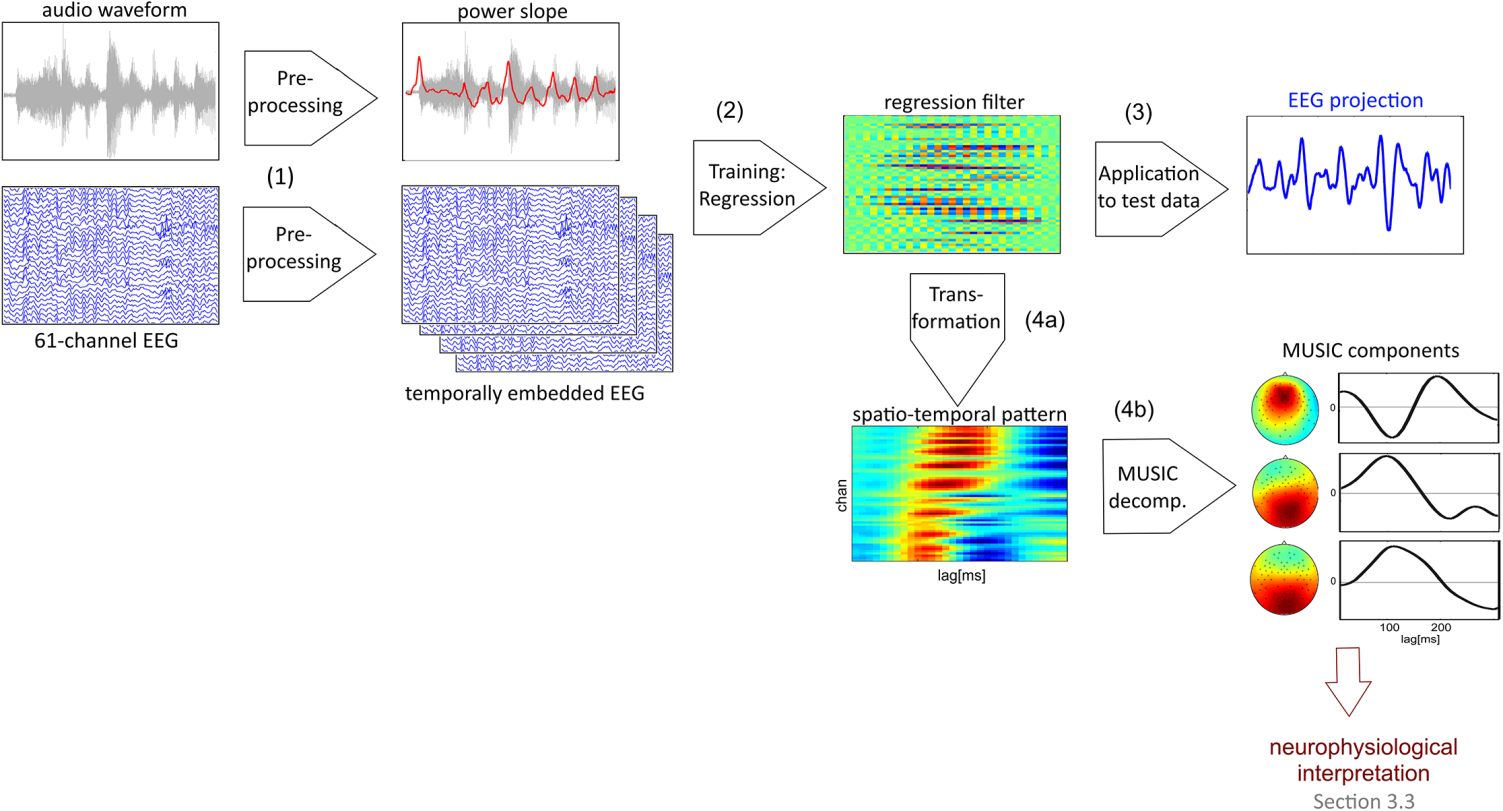
EEG feature extraction. (1) In the first step of the analysis the 61-channel EEG signal (after generic preprocessing, see Methods) is temporally embedded and the power slope of the audio signal is extracted. In the training step (2) the embedded EEG features are regressed onto the audio power slope (Ridge Regression). After that (3) the resulting spatio-temporal filter (regression weight matrix) reducing the multichannel EEG to a one-dimensional projection is applied to a new presentation of the same stimulus. The regression filter can be transformed (4a) into a spatio-temporal pattern that indicates the distribution of information which is relevant for the reconstruction of the audio power slope. This spatio-temporal pattern, in turn, can be (4b) decomposed into components (derived with the MUSIC-algorithm) which have a scalp topography and a temporal signature. The EEG projections obtained in (3) subsequently are examined with respect to Cortico-Acoustic correlation (CACor).

The analysis aims to obtain a representation from the ongoing EEG that reflects brain responses to the onset ‘landscape’ of a music stimulus. It can be divided into four modules for (1) preprocessing EEG and audio data, (2) calculating spatio-temporal regression filters for optimally extracting EEG features, (3) applying the derived filters to new data in order to extract EEG projections, and (4) transforming the spatio-temporal filters into a representation suitable for neurophysiological interpretation. Finally, the synchronization between the extracted EEG projections in (3) and the audio stimulus is examined at several levels of temporal resolution and the results are related to behavioral results and stimulus characteristics. Fig 2 summarizes steps (1) to (4).

ERP responses are phase-locked to rapid intensity changes that indicate tone onsets in the music stimulus. In order to extract a component from the ongoing EEG that optimally reflects onset-related brain responses we apply Linear Ridge Regression [46] to maximize the correlation between EEG and the audio power slope of the stimulus, an acoustic feature that represents the intensity changes that are expected to trigger ERP responses.

1. Pre-processing of EEG data: temporal embedding
  Since it is not clear a priori by how much the EEG response lags behind the presented stimulus, we perform a temporal embedding of the EEG signal, as proposed in [47], which allows to deal with relationships between signals with unknown delay: to the EEG data matrix X of m observations x n channels additional dimensions that are copies of X, time-shifted by 1,…, 30 data points are added as ‘artificial’ channels, resulting in a data matrix of the dimensionality m x 31. This allows to capture brain responses within a latency of 0 to 300 ms. The audio power slope is extracted as described above.
2. Training: Calculation of spatio-temporal regression filters
  We train Linear Ridge Regression models to optimize the correlation between the temporally embedded EEG of single subjects and the power slope of the audio signal. This is done in a leave-one-recording-out cross-validation approach. For a given stimulus and subject a spatio-temporal filter is trained on the concatenated EEG of two presentations (training set) and applied to the EEG of the remaining third presentation (test set). This is repeated three times in all possible combinations, so that each presentation is once the test set. The temporal embedding performed in step 1 blows up the dimensionality of the EEG signal from 61 to 61 x 31 ‘channels’ and strongly requires regularization. Therefore, instead of using the standard Ordinary-Least-Squares solution for Regression, we apply Ridge Regression where regularization is introduced by adding a ‘ridge’ to the empirical covariance matrix according to an analytically determined shrinkage parameter [48]. This is a common technique to stabilize the estimation of covariance matrices [49].
3. Application of regression filters to new data
  The cross-validation procedure described above resulted in a one-dimensional EEG projection for each stimulus presentation, and, accordingly, in 27 EEG projections for one stimulus (for nine subjects and three presentations each). This set of EEG projections served as basis for examining the relation between brain signal and onset structure at several levels.
4. Transformation and decomposition of spatio-temporal filters
  The spatio-temporal filters that result from step 1 are matrices of the dimensionality n channels x t time lags (here: 61 channels x 31 time lags = 1984 dimensions) which correspond to a sequence of scalp topographies. However, these maps are not appropriate for neurophysiological interpretation; see [50] for a detailed discussion. Instead, the filter maps need to be transformed into patterns (see transformation (4a) in Fig 2) which specify how the activity of the respective sources in brain is projected on the scalp (see [51] for details on the underlying forward model of EEG generation and [50] for the details of the actual transformation). This allows to examine how the information that is used to reconstruct the power slope of the stimulus is distributed in space (on the scalp) and time (relative to the stimulus). An example of such a spatio-temporal pattern is given in the upper panel of S1 Fig. Interpreting the matrix representation of this information is not very intuitive. Therefore, it is desirable to distill a representation from the spatio-temporal regression patterns that is reduced in dimensionality and that has a form that allows for a better comparison with conventional ERP components, e.g., consisting of one or more 'components' with a spatial pattern and a time course.

In the following, we describe such a technique that is based on Singular Value Decomposition (SVD) which is a standard method for expressing the characteristics of a matrix by means of its eigenvectors and -values. Eigenvectors are orthogonal to each other. It is, however, questionable that neural sources and their physiological patterns should obey an orthogonality constraint. Therefore, we apply a variant of the the MUltiple SIgnal Classification (‘MUSIC’) algorithm [52] to extract physiologically plausible estimates of neural components that have produced the subspaces which contain the characteristic features of the spatio-temporal pattern matrices, but are not necessary orthogonal. This transformation corresponds to (4b) in Fig 2.

The 61 x 31 dimensional matrices were factorized using Singular Value Decomposition (SVD) and subsequently reduced to a lower-dimensional subspace covering a predefined portion (98%) of the variance. To the spatial SVD components the MUSIC algorithm is applied in order to extract a new set of (non-orthogonal) patterns. The variant of the MUSIC algorithm used here finds a set of dipoles that, according to pre-defined standard head model and a least-squares metric, match the observed patterns best. These dipoles can be projected out to results in a set of (non-orthogonal) spatial MUSIC components, in our case 2–4 components per subject and stimulus. Furthermore, corresponding time courses can be determined based on the original SVD factorization. In summary, the MUSIC procedure achieves a dimension reduction and decomposition of the spatio-temporal regression patterns into a set of dipole-source related scalp topographies with corresponding time courses. The lower panel of S1 Fig shows three spatial and temporal MUSIC components that were derived from a spatio-temporal matrix (upper panel) by means of the MUSIC algorithm.

Comparing these decompositions between subjects is difficult, since no canonical representation can be derived. Visual inspection of this large collection of scalp patterns suggested the presence of a scalp pattern that was consistent between subjects and stimuli and resembled the scalp topographies of the onset-related ERPs for Chord sequence. In the decomposition this spatial component occurred either as a first, second or third component. Since the present analysis is interested in brain responses to tone onsets we extracted a reference pattern from the onset-related ERPs obtained by classical ERP analysis for Chord sequence. This was done by averaging the scalp topography of all subjects within a 20ms time window that corresponds to the maximum amplitude of each subject’s onset ERP at channel Fz. The time windows were determined manually and ranged between 130 ms and 280 ms after tone onset. For the results of the ERP analysis and the reference pattern see Section ‘Interpretation of spatio-temporal patterns’. Subsequently, the distance between all spatial components and the reference pattern was calculated and the most similar pattern for each subject and stimulus was selected for further comparison. In 43% (38%, 18%) of the components the selected pattern occurred in the first (second, third) MUSIC component.

#### EEG Analysis: Applying Cortico-Acoustic Correlation

In a first step we calculate the global correlation coefficient between EEG projection and the audio power slope, termed Cortico-Acoustic Correlation (CACor): We determine the CACor coefficient for each single presentation (nine stimuli, nine subjects, three presentations per stimulus and subject) and assess its significance (details as described below). This aims at assessing whether the onset structure of the stimulus is significantly reflected in the brain signal. In S2 Fig this step of analysis corresponds to the ‘presentation level’. In addition, the Grand Average of all 27 EEG projections per stimulus is calculated and a group level CACor coefficient is derived for each stimulus.

Subsequently, the relationship between CACor, behavioral results and stimulus properties is compared between stimuli (see column ‘Between stimuli’). Based on the significance of CACor coefficients for each single presentation a global CACor score is calculated for each stimulus that specifies in how many of the 27 presentations a significant CACor is present. These can be summarized in a CACor score profile for the nine stimuli. In a similar fashion, a Coordination score profile is constructed from the Coordination scores derived in the behavioral experiment (see above). Additionally, for each of the nine acoustic/musical properties that were obtained in the audio analysis a profile that describes the magnitude of the respective stimulus feature for all stimuli was constructed. The pairwise correlation between CACor score profile, Coordination score profile and music feature profiles is calculated.

In a second step the dynamics of the brain-stimulus synchronization within each stimulus is examined by calculating a group-level CACor in a time-resolved manner (see also S2 Fig rightmost column): For each stimulus the 27 EEG projections (three for each subject) are averaged and segmented into 90% overlapping time frames of 3 s duration. Subsequently, CACor is calculated for each time frame, resulting in a time course of correlation coefficients that has a sampling rate of 3.3 Hz. Correlation coefficients between this time course and the time courses of the nine acoustic/higher-level music features are determined. In an analogue manner, the relation between mean tension ratings and music features is determined. S2 Fig gives a complete overview about data types, features and correlation coefficients that are calculated in the present analysis.

#### Significance of correlation

In the present analysis we encounter two statistical problems that are typical for correlation-based analysis of time series data related to naturalistic music stimuli:

First, both, the EEG signal and the audio power slopes (and also other features derived from the music stimuli) contain serial correlation, i.e., subsequent samples are not independent of each other. Thus, the assumptions underlying standard tests for significance of correlation are not satisfied. This can be accounted for in different ways: Pyper et al. [53] propose a method that estimates the effective degrees of freedom based on the cross-correlation between the two respective time courses and, then, calculate p-values based on the corrected degrees of freedom. In the music domain this has been applied in [33]. A different strategy can be pursued by applying randomized permutation tests with surrogate data as proposed in [54] and described for a music-related analysis in [37]

Notably, in the present context, both methods have some specific drawbacks. Pyper et al.’s method ‘punishes’ cross-correlation between two time series in order to avoid inflated correlation coefficients—a technique that also reduces the sensitivity. Permutation tests, in principle, can be regarded as more sensitive, but permutation tests in combination with temporal embedding may be problematic. The opportunity to account for a delayed relationship between both signals that is given by the temporal embedding can, dependent on the relation between window length of embedding and structure of the target function, severely affect the sensitivity of the permutation test.

In order to gain more insight with respect to this aspect, we assessed the significance of CACor at single subject and single presentation level using both methods. For Pyper et al.’s method the maximal time lag of autocorrelation that was taken into account was 2 s.

As a second statistical problem a complex auditory stimulus comprises a multitude of features that are not independent of each other, but are correlated to different degrees. For the present selection of stimuli the correlation coefficients ranged between r = -0.74 and r = 0.93. To account for this correlation, the relation between CACor time courses/tension ratings and music features was determined using the partial correlation coefficient [55]. This *post-hoc* approach is a way to exert statistical control over variables in a setting where experimental control on the different aspects that are to be investigated is ruled out by design and has been applied in a similar context before [37]. For assessing the significance of partial correlation coefficients, however, the permutation testing approach is not optimal, since it is not possible to generate surrogate data with the same intrinsic relations as the extracted music features. Therefore, Pyper et al.’s method (maximal lag 10 s) is applied to correct for autocorrelation in order to estimate the significance of partial correlation coefficients. The resulting p-values were corrected for multiple comparisons (false discovery rate (FDR), *q* < 0.05).

## Results

### Tension ratings

**Fig 3:**
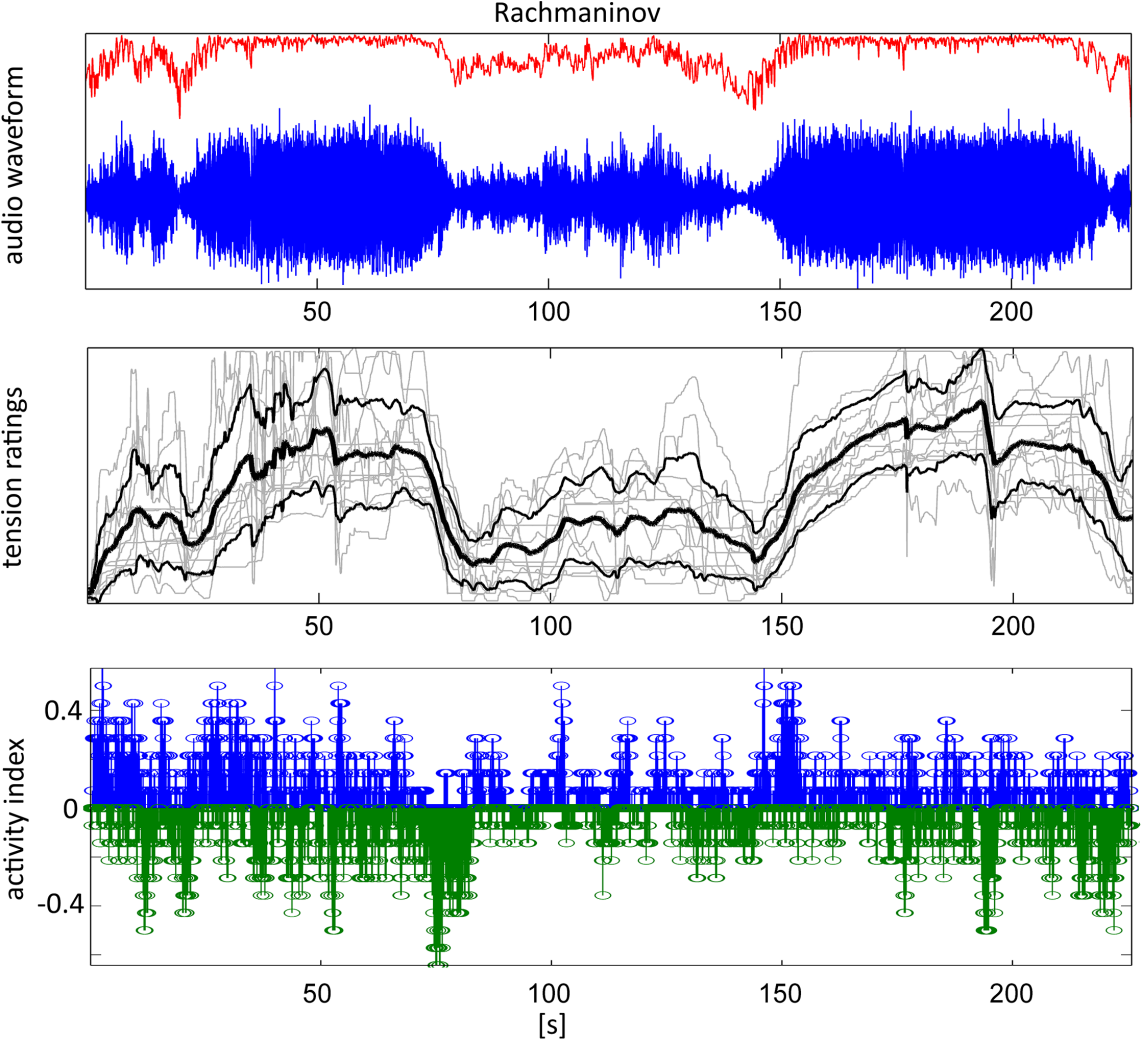
Example of stimulus waveform and tension ratings. Top: Audio signal (blue) and sound intensity (red) for the Rachmaninov Prelude (entire stimulus). Middle: Tension ratings of single subjects (grey, N = 14), Grand Average (black, thick line) and standard deviation (black, thin line). Bottom: Activity index indicating the percentage of rising (blue) or falling (green) tension ratings at a given time point.

Fig 3 gives an example of the continuous tension ratings obtained in the behavioral experiment. The upper panel shows the audio waveform (blue) of the entire stimulus (Rachmaninov Prelude No. 5) and the sound intensity (red). In the lower panel the individual tension ratings are plotted along with the Grand Average tension ratings. The bottom panel shows the activity indices that indicate how consistently tension ratings rise/fall within the group of subjects at a given time point. While the individual tension ratings vary considerable between subjects, the Grand Average and the results of the activity analysis show clearly that the three-part macro-structure contained in time course of the sound intensity is represented in the tension ratings.

### Cortico-Acoustic Correlation

#### Cortico-Acoustic Correlation for single presentations

**Fig 4:**
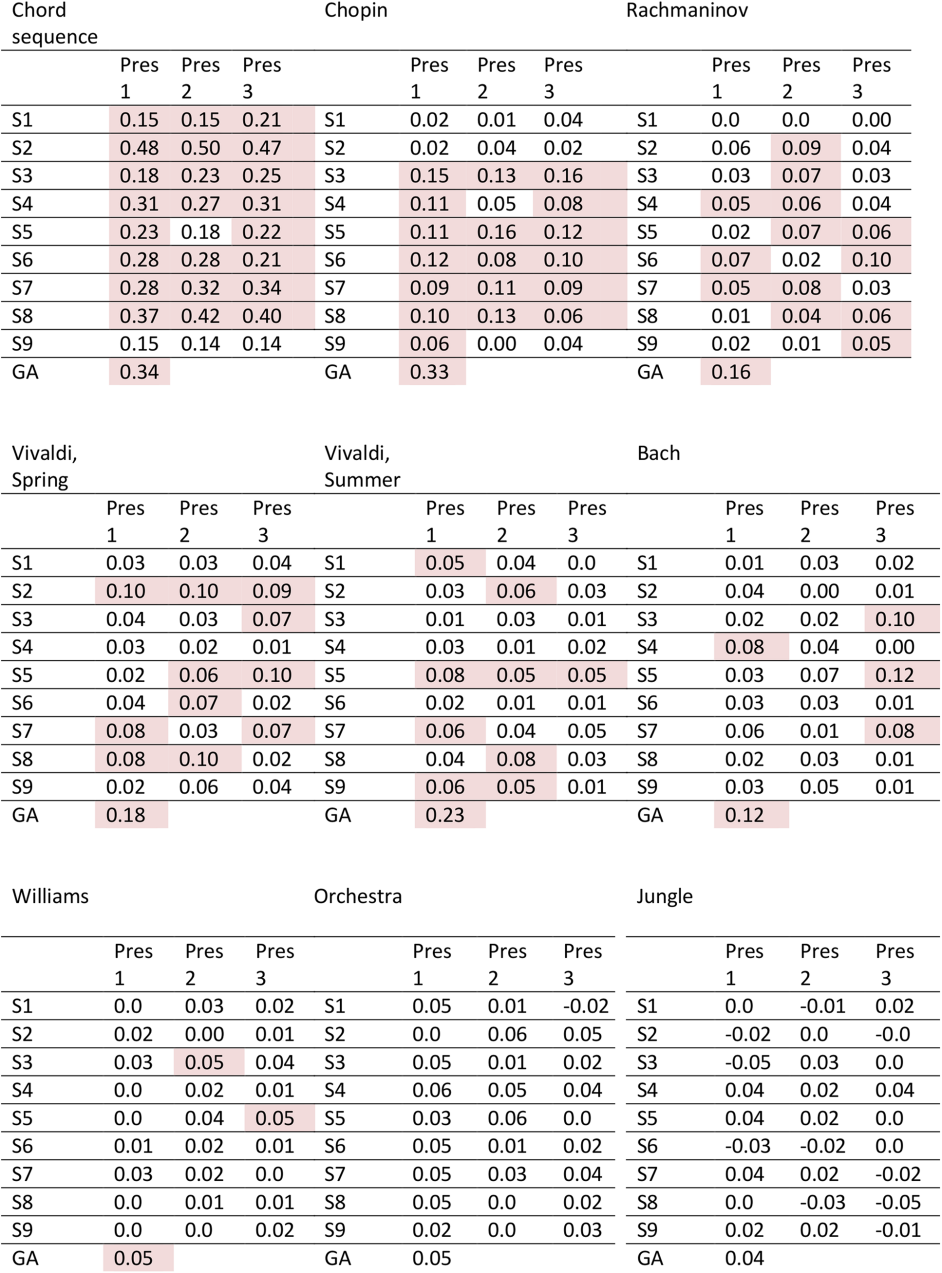
Table of CACor coefficients. CACor coefficients for each single subject and each stimulus presentation; GA: Grand Average (N = 27). Pink shading indicates significant positive correlation between EEG projection and audio power slope. Significance was determined by applying permutation tests and subsequently performing Bonferroni-correction for N = 27 presentations per stimulus.

**Fig 5:**
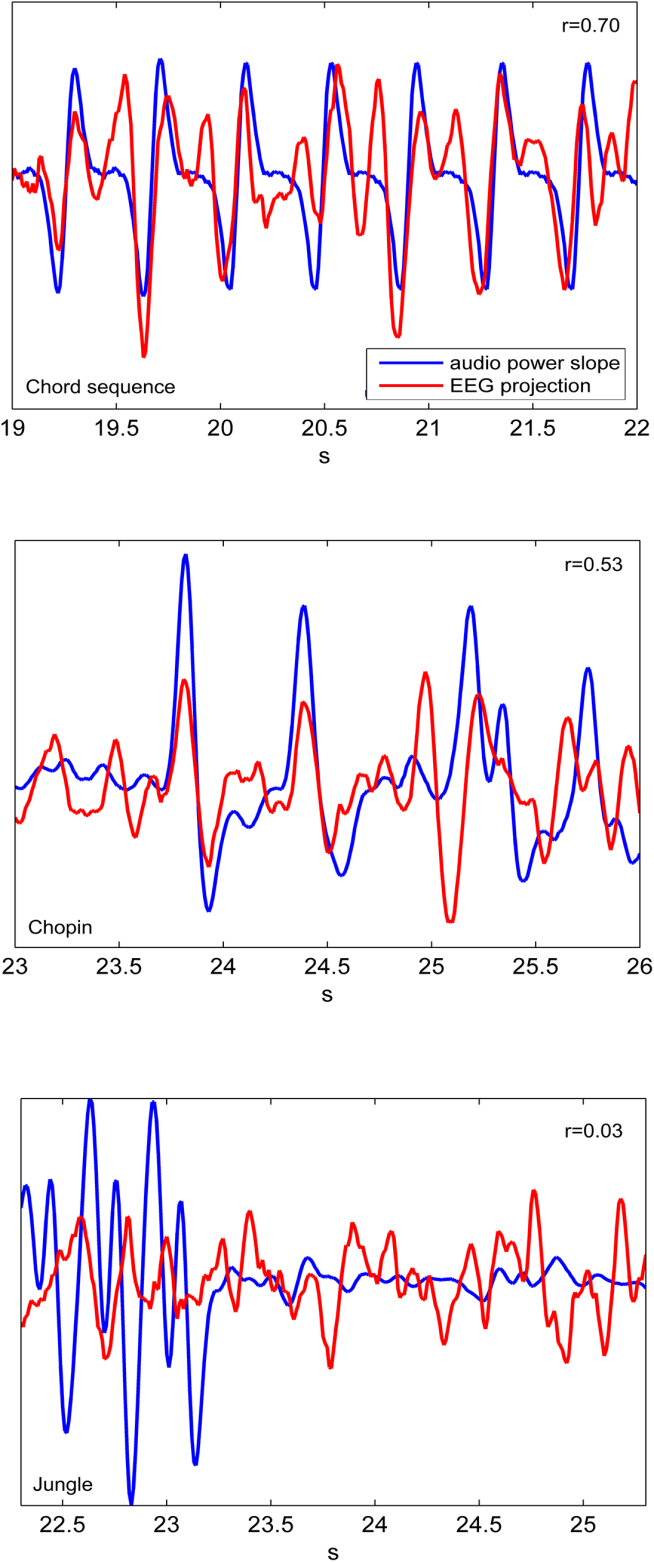
EEG projections. Three examples that show 3s-long segments of an extracted EEG projection (red) for a single stimulus presentation and a single subject and the respective audio power slope (blue) of Chords, Chopin Etude and Jungle noises. Note that in the optimization procedure a time lag between stimulus and brain response is integrated in the spatio-temporal filter, and that, consequently, the resulting EEG projections shown here are not delayed with respect to the audio power slope. The correlation coefficients indicate the magnitude of correlation for the shown segment of 3 s.

**Fig 6:**
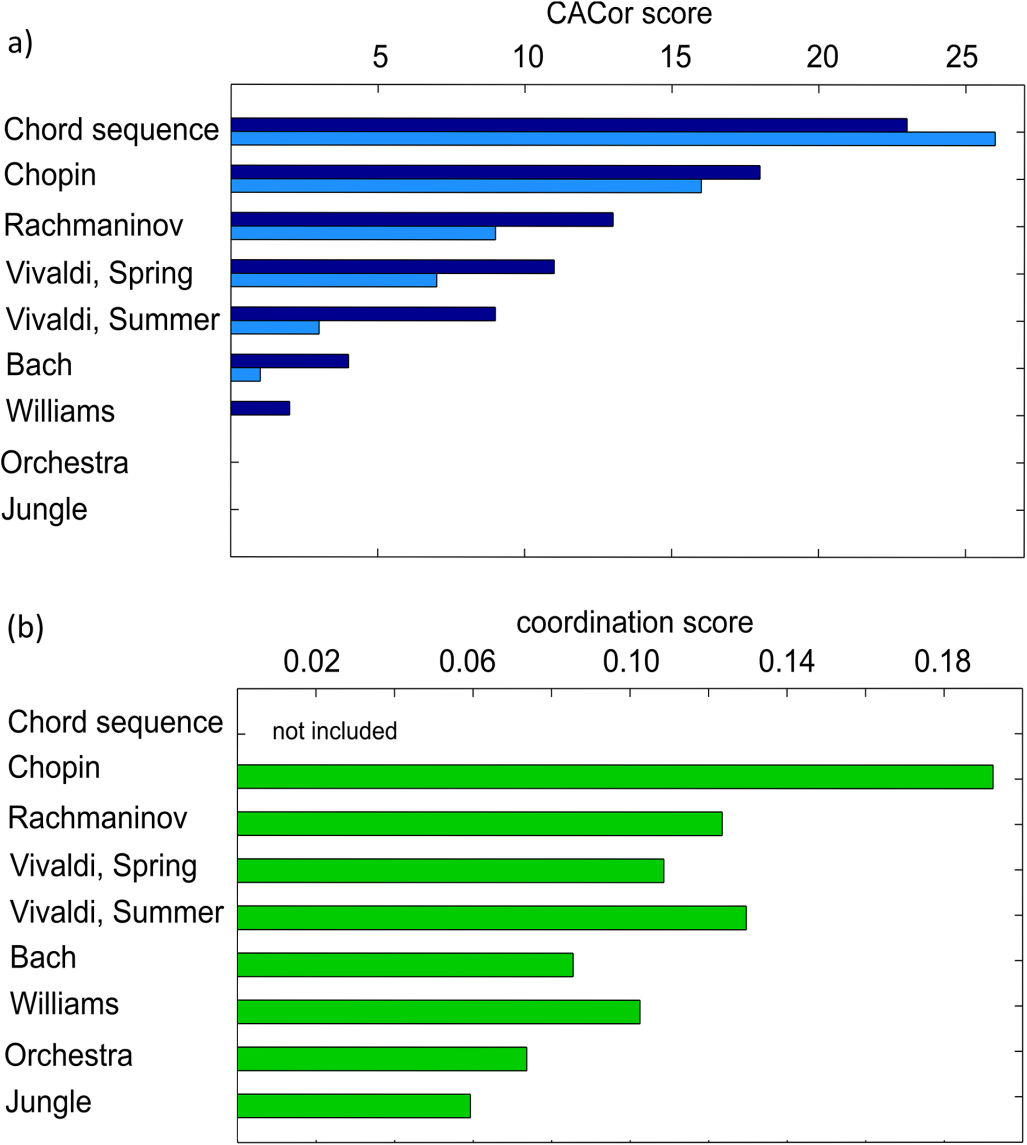
CACor score profile and Coordination score profile. The CACor score profile for the set of nine stimuli summarizes in how many of the 27 presentations significant Cortico-Acoustic Correlation was detected. Significance of correlation was determined (1) in a permutation-testing approach (darkblue bars) and (2) with Pyper et al.’s method (light blue bars) to estimate the effective degrees of freedom (see Methods). b) Behavioral Coordination score profile. All profiles are sorted according to the descending CACor score.

The table depicted in Fig 4 gives CACor coefficients for each single subject and each stimulus presentation. The bottom line of each table contains the correlation coefficient for the Grand Average EEG projection (average of the 27 EEG projections for each stimulus). Note that correlation coefficients cannot be compared between stimuli, since duration and autocorrelation properties differ between stimuli. Shaded cells indicate significant correlation at the level of alpha = 0.05. Significance was determined by applying permutation tests and subsequently correcting for the number of 27 presentations per stimulus (see Methods, Subsection ‘Significance of Correlation‘). Stimuli were ordered according to the total number of presentations with significant CACor (called CACor score in the following). Since these were derived in a cross-validation approach (see Methods, Subsection ‘EEG analysis: Calculating Cortico-Acoustic Correlation‘) significant correlation coefficients can be regarded as reflecting a genuine influence of the stimulus on brain responses that generalize across several presentations of a stimulus. To give an impression of the extracted EEG projections and their relation to the audio power slope three examples are shown in Fig 5. Fig 6A summarizes the corresponding CACor scores into a CACor score profile for the set of nine stimuli. For comparison, both, the CACor score profile that was derived by applying permutation tests (dark blue bars), and that derived by applying Pyper et al.’s method [53] to assess the significance of correlation in signals containing serial autocorrelation (light blue bars) are given. Although there are differences in the absolute CACor scores for both methods, the ranking of the stimuli does not change. Importantly, the comparison shows that the zero scores for Orchestra and Jungle indicate an absence of significant CACor for these stimuli and are not introduced by the permutation test approach. In the following the profile derived by permutation tests is used for further analysis.

Stimuli differ strongly with respect to the consistent presence of significant CACor for the 27 presentations with a CACor score of 24/27 (89%) presentations for Chord sequence, 18/27 (67%) presentations for Chopin, 13/27 (48%) presentation for Rachmaninov, 11/27 (41%) presentations for Vivaldi, Spring, 9/27 (33%) presentations for Vivaldi, Summer 4/27 (15%) presentations for Bach, 2/27 (7%) presentations for Williams and 0/27 (0%)presentations for Orchestra and Jungle. In the Grand Average CACor is significant for Chord sequence and all music pieces, but not for Orchestra and Jungle.

Subjects differed with respect to the total number of presentations with significant correlation, scoring between 4/27 presentations (S1, S9) and 15/27 presentations (S5). With 25/81 presentations with significant correlation for first presentation, 27/81 for the second presentation and third presentation no influence of repetition of the stimuli was found.

#### CACor, tension ratings, and stimulus features

Fig 6B compares the behavioral Coordination score profile for the nine stimuli with the CACor score profile. Note that the regular stimulus chord sequence was not included in the behavioral experiment. However, in the CACor score profile blank bars for Orchestra and jungle denote a score of zero. Table 1 gives correlation coefficients (Spearman’s rho) that quantify the correlation of (a) CACor score profile and (b) Coordination score profile with music feature profiles that relate to nine stimulus features. Each of these is represented by scores that indicate the average magnitude of a specific acoustic/higher-level musical property for the nine stimuli. S3 Fig gives a detailed overview how stimuli differ with respect to each of the nine acoustic/higher-level musical properties. The bottom line of Table 1 contains the correlation between CACor score profile and Coordination score profile. The CACor score profile is significantly positively correlated with the Sharpness profile and significantly negatively correlated with the Spectral centroid profile. Furthermore, moderate, but non-significant negative correlation of the CACor score profile with the Fluctuation entropy profile is present. None of the correlation coefficients between Coordination score profile and music feature profiles reached significance. CACor score profiles and Coordination score profiles are significantly correlated with r = 0.9, p = 0.005.

**Table 1:** Relation between CACor scores and music features. Spearman’s correlation coefficient (a) between CACor score profile and music feature profiles for nine acoustic/higher-level music features, (b) between Coordination score profile and music feature profiles. Bottom line: Correlation between CACor scores and Coordination scores. Asterisks indicate significant correlation.

	a)	b)
Stimulus feature	CACor score	Coordination score
Sound intensity	0.24	0.0
Sharpness	0.71*	0.57
Spectral centroid	-0.69*	-0.57
Spectral entropy	-0.52	0.23
Spectral flux	0.28	0.11
Fluctuation centroid	0.09	-0.14
Fluctuation entropy	-0.65	-0.19
Pulse clarity	-0.32	-0.02
Key clarity	0.58	0.38
Coordination Score	0.90*	

#### Dynamics of CACor

We examined how changes in group-level CACor (correlation between the Grand Average EEG projections and audio power slope) relate to changes in stimulus features and in mean tension ratings during the course of a stimulus. Note that in this part of the analysis correlation coefficients indicate whether CACor and a particular property of the music stimulus co-vary, but do not necessarily inform about significance of global CACor. The full set of partial correlation coefficients (see Section ‘ EEG Analysis: Applying Cortico-Acoustic Correlation’ for details of the calculation) is given in S2 Table. Of these, only a small number of correlation coefficients were found significant, most consistently we found a significant positive correlation of mean tension ratings with Sharpness and a negative correlation with Spectral Entropy.

Out of the nine (eight for the tension ratings, respectively) stimuli the romantic piano pieces (Chopin and Rachmaninov) show the clearest relations between CACor and music features, followed by both Vivaldi stimuli and, then, by one of the non-music stimuli. A similar trend is visible for the relation between tension ratings and music features.

### Interpretation of spatio-temporal patterns

**Fig 7:**
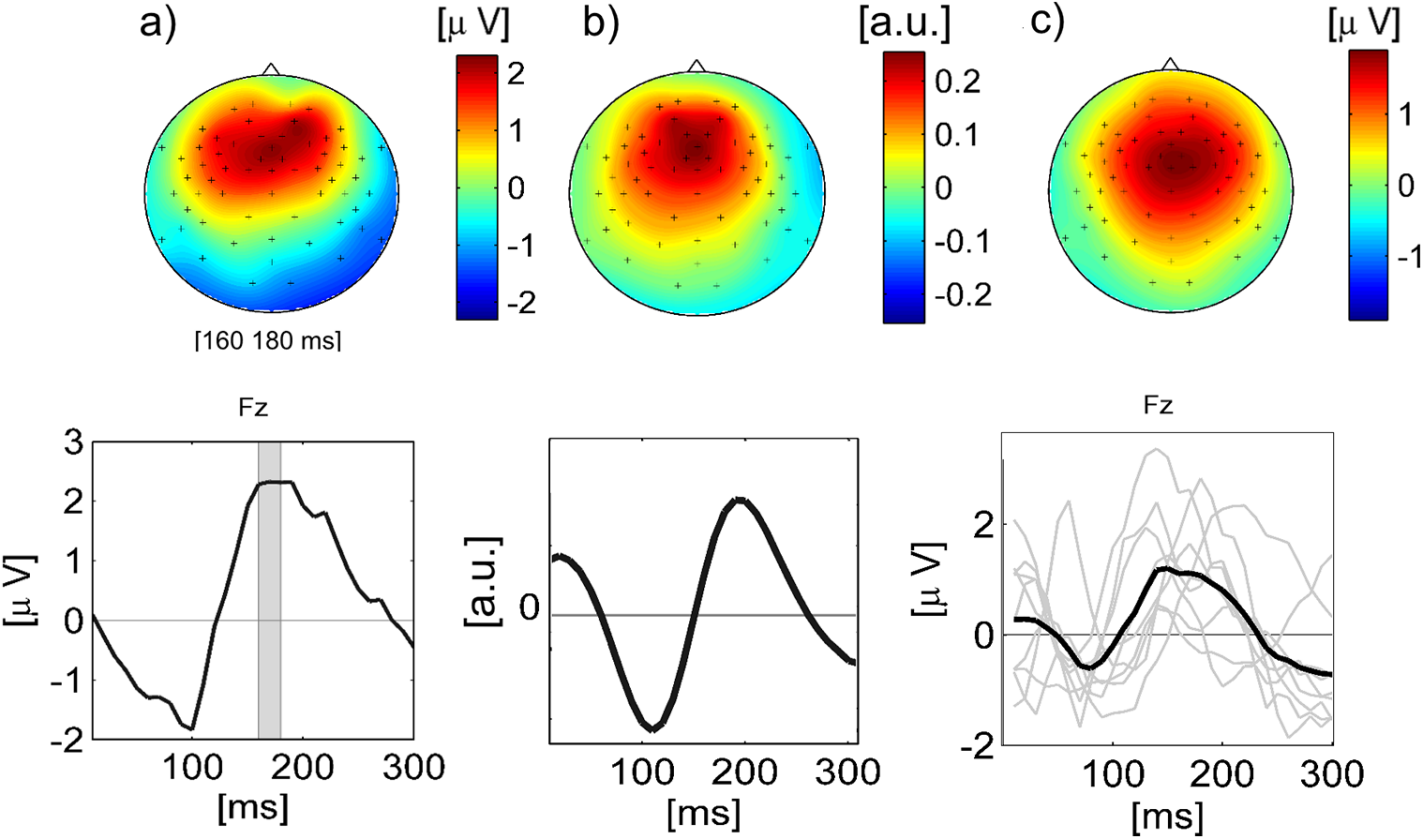
ERPs and MUSIC components. (a): Scalp topography (top) and time course (bottom) of ERP (single subject) derived by averaging channel Fz for all tone onsets of the stimulus 'Chord sequence'. The scalp topography corresponds to the shaded interval of 160–180 ms in the time course. (b): Spatial (top) and temporal (bottom) dimension of MUSIC component derived from spatio-temporal patterns for the same subject. c) Top: Reference pattern for selection of consistently occurring MUSIC components. This pattern is the Grand Average scalp topography obtained with classical ERP analysis. Individual scalp patterns that are contained in the Grand Average were determined by taking the mean scalp pattern across a 20 ms window that corresponds to the maximum amplitude of each subject’s onset ERP at channel Fz. The time windows of maximum amplitude were determined by visual inspection and ranged between 130 and 250 ms after tone onset. The bottom part of c) shows the individual time courses (grey) as well as the average time course (black).

Fig 7A shows the scalp topography and time course of the ERP that were derived by classical averaging techniques from the EEG data of a single subject (S2) for the Chord sequence (see Methods, Subsection ‘Stimuli’). The upper part shows the scalp topography of the time interval of 160–180 ms after chord onset. Below, the time course of EEG channel Fz for the first 300 ms after chord onset is depicted. In Fig 7B the spatial (top) and temporal (bottom) dimension of one of the MUSIC components for the same subject are shown. Both scalp topographies show a positive fronto-central distribution. In the time course of the ERP a negative deflection at 100 ms is followed by a peak at appr. 170 ms, while the temporal dimension of the MUSIC component is represented by a negativity at 110 ms that is followed by a positive peak at about 200 ms. The upper part of Fig 7C shows the scalp topography that was used as a reference to select one MUSIC component per subject and stimulus for comparison. It is represented by the average scalp pattern across subjects for the Chord sequence in a time interval of 20 ms around the individually determined point of maximum amplitude within the range of 150 ms to 230 ms after tone onset (for details see Methods). The bottom part of Fig 7C shows corresponding individual time courses for all subjects at channel Fz (grey) as well as the Grand Average time course (black).

**Fig 8:**
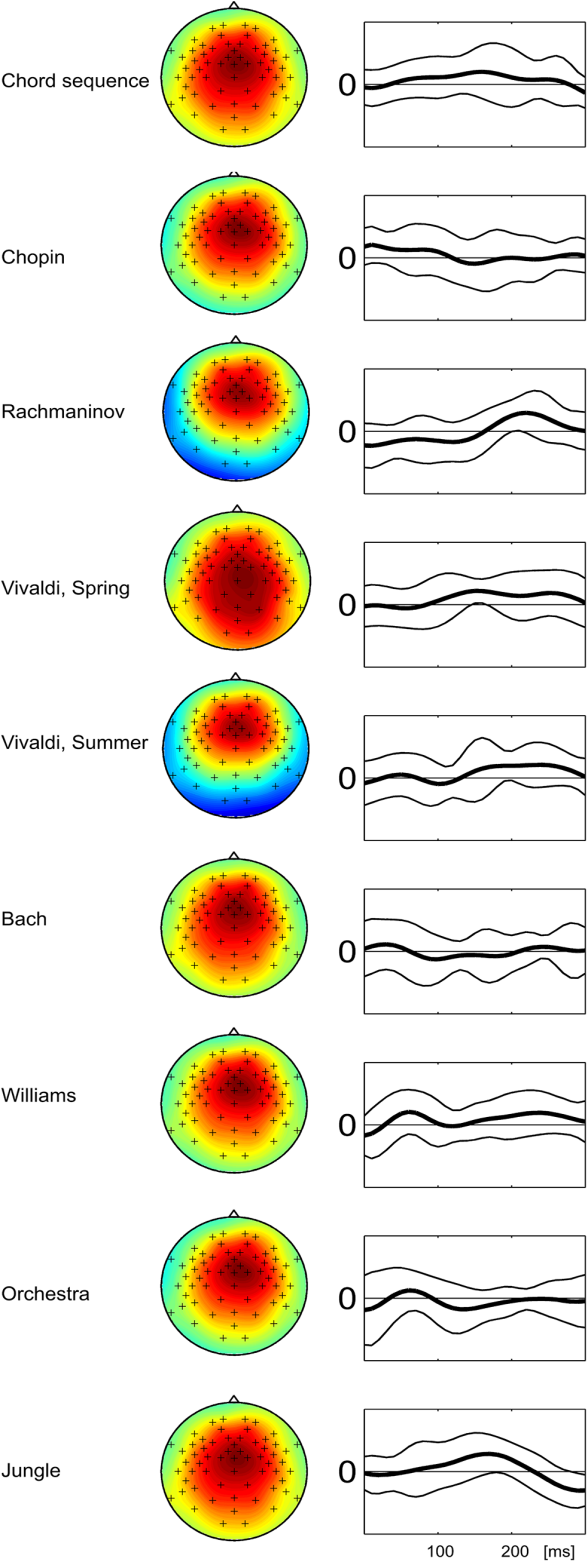
MUSIC components (Grand Average) for all stimuli. Scalp topographies and time courses (thick line: average of all subjects, thin lines: standard deviation) of the MUSIC component that was extracted most consistently from the decomposed spatio-temporal patterns. Single-subject scalp topographies that are included in these averages are contained in the Supplementary Material.

Fig 8 shows the spatial and temporal dimensions of the selected MUSIC component for the nine stimuli, averaged across subjects. A complete collection of the individual scalp patterns (one for each stimulus and subject) is contained in S4 Fig. Note that these patterns were selected for maximal similarity with a reference pattern as described above, and, therefore, necessarily are similar to some extent. The averaged scalp topographies for all nine stimuli show a positive fronto-central pattern. The temporal patterns are much more variable.

## Discussion

The present study demonstrates that multivariate regression-based methods for EEG analysis can extract neural responses to acoustic onset patterns at the level of single subjects and single presentations of complex original music stimuli. Our results were obtained in an experimental setup (see Fig 1 for an overview) where stimuli from different sound categories were presented, ranging from simple tone sequences to romantic piano music, including also non-musical complex sounds. EEG was recorded from nine subjects that listened to these stimuli. In a separate experiment continuous tension ratings were recorded from a group of 14 different subjects. In the audio analysis part a set of nine acoustic/musical features was extracted from the waveform of each stimulus.

From the multi-channel EEG a projection was derived that optimally represented the onset-related brain responses. Subsequently, the Cortico-Acoustic Correlation (CACor) between EEG projection and audio power slope was determined as a measure of brain-stimulus synchronization. From the tension ratings a behavioral Coordination Score was obtained for each stimulus (see Section ‘Preprocessing of tension ratings‘, for an overview on the features that were extracted from the EEG and from the tension ratings, see S2 Fig).

The nine stimuli differed with respect to the frequency of occurrence of significant CACor in single stimulus presentations. The stimuli with consistently occurring CACor also produced strongly coordinated tension ratings in the behavioral experiment. The results of the audio analysis part helped to attribute this finding to the stimulus feature Sharpness and to tempo-related and spectral features. At a finer time resolution CACor and tension ratings were modulated by similar music features, but in different ways, i.e., increasing Sharpness co-occurred with decreasing tension.

### Method

Our results demonstrate that the proposed regression-based method allows to extract a neural representation of the sequence of note onsets that constitute a complex naturalistic stimulus at the level of single presentations and single subjects. Our stimulus set contained six original pieces of music, a simple chord sequence with repetitive structure, and two non-musical complex stimuli. In all but the non-musical stimuli (Orchestra and Jungle) the note onsets structure of the stimulus was reconstructed from the EEG with significant correlation at least once at single-presentation level and also from the Grand Average EEG features (see Results, Subsection ‘Cortico-Acoustic Correlation for single presentations’). On the one hand, this shows that the audio power slope captures features of the music stimulus that have a marked influence on the EEG signal. On the other hand, it demonstrates that Ridge Regression with the audio power slope as a target function can extract a component from the listener’s EEG signal that significantly reflects an acoustic aspect of a naturalistic music stimulus. By following a cross-validation approach we demonstrated that this relationship between EEG and stimulus reflects genuine stimulus-related activity in the listener’s EEG that generalizes across presentations of the same stimulus. Our results help to delineate onset-related brain responses as one distinct component of the ongoing EEG signal of subjects who listen to complex music. This insight complements previous approaches that aimed at decomposing ongoing EEG with respect to stimulus structure [16] and [15], [4], and, therefore, adds to the growing body of knowledge about how a naturalistic complex music signal is represented in the brain.

While it has been established for a long time that rapid changes in sound intensity generate phase-locked neural responses [2], (to our knowledge) integrating the power slope of an audio signal in the optimization process for extracting these brain responses from the ongoing EEG represents a novel approach. Prior to the present analysis it has been applied in [56] to a set of semi-musical repetitive monophonic and polyphonic sound patterns in order to investigate auditory stream segregation. The present analysis represents the first attempt to apply this method in an experimental paradigm where subjects listen without any task to stimulus material that is comparable to music/non-musical sounds that we consume in everyday life.

In a comparable setting Jäncke et al. [7] have shown that the relationship of band-power modulations in several frequency bands with the stimulus envelope (as well as with a measure of acoustic complexity) is highly variable across presentations of the same stimulus. At a global level, however, the mental state of listening to music can be characterized consistently by increases in band-power several bands. These results were interpreted as pointing to the non-stationary and dynamic nature of the neurophysiological activations related to music listening. Our results complement these findings as they demonstrate that a consistent relationship between brain signal and stimulus structure can be established if instead of band-power features the broadband EEG is examined. Vice versa, the variability of band-power modulations demonstrated in [7] may explain some of the unresolved variance in our results, since an ‘obligatory’ representation of onsets in the EEG may be obscured by the non-stationarity in band-power. In combination, the present results and that of Jäncke et al. suggest that two complementary avenues for research exist for investigating natural music with EEG. On the one hand, the broadband EEG can give valuable insight into the representation of the (invariant) stimulus structure in the brain signal, an approach that can be summarized as ‘looking for the common features between subjects’. On the other hand, band-power modulations that, putatively, may be related to different individual processes may, in principle, inform on the sequence of brain states that characterizes a unique listening experience. This approach can be summarized as ‘examining what makes a listening experience individual’. Here, however, pinpointing band-power fluctuations to a specific aspect of listening is an experimental challenge that is yet to be addressed. In summary, combining both approaches may provide for a comprehensive analysis integrating fixed stimulus-driven and non-stationary individual aspects of cerebral music processing.

Linear Ridge Regression with the audio power slope as a target function produces spatio-temporal filters that reduce the multichannel EEG to a one-dimensional time course that represents the brains reaction to sound onsets with enhanced signal-to-noise ratio while preserving the EEG’s exquisite time resolution.

The proposed analysis pipeline is assembled from well-established methods with relatively simple underlying ideas, such as Ridge Regression, Single Value Decomposition (SVD) and Least-Squares Optimization. Our results, however, show that enriching these ‘old’ mechanisms with specific extensions allows deriving significant results at single-trial level in a challenging setting. In particular, the combination of multivariate EEG analysis (that in itself is highly effective for enhancing the signal-to-noise-ratio) with temporal embedding adds to the strength of the method as it enables fine-tuning spatio-temporal filters to stimulus characteristics and to individual differences in latencies of brain responses. Crucial for the success of this approach, however, is appropriate regularization. Furthermore, relating brain responses to a stimulus’ power slope (instead of the sound envelope that numerous approaches focus on) exploits the brain’s sensitivity to change.

Technically, the application of this method is not restricted to a particular type of auditory stimulus, since the power slope can be derived in a simple procedure from any audio waveform. In principle, this technique may be helpful in a range of scenarios related to onset-ERPs as it mitigates the demand for a high number of trials that is typical for EEG averaging techniques. It is applicable at single-subject and single-presentation level and it is appropriate for complex long stimuli. Since in the extracted EEG projections the time-resolution of the EEG is preserved, this method allows for subsequent investigations at several time scales. Therefore, the present approach represents an important contribution to the broad range of methods that have been proposed in auditory processing research in order to investigate the relation between envelope-related features of sounds and brain signals, such as auditory-evoked spread spectrum analysis (AESPA) [13], convolutional models [11], cross-correlation analysis [6], [11] or Cortico-Acoustic coherence [30]. It may open up a new avenue for transferring findings that have been made with respect to simple stimuli and at group level to the level of subject-individual analysis and towards more naturalistic stimuli.

#### Neurophysiological interpretation

The proposed regression-based method not only derives a condensed and optimized representation of the multi-channel EEG that subsequently can be examined at several time scales. It also allows to learn about the temporal and spatial distribution of information in the EEG signal that is relevant for the optimization process.

For each subject and stimulus spatio-temporal patterns derived from the regression filters were decomposed into several components (called MUSIC components according to the decomposition algorithm) with a temporal and spatial dimension each (see Methods, Subsection ‘EEG analysis: Calculating Cortico-Acoustic Correlation‘). To obtain a common basis for comparison in this large set of scalp topographies we selected the MUSIC component with the scalp topography that is most similar to a reference topography that was derived by averaging EEG data for all tone onsets of the stimulus Chord sequence. This onset-related ERP (see Fig 7) was fronto-centrally distributed with a negative deflection at appr. 100 ms after tone onset, followed by a positive peak between 150 and 200 ms after tone onset and, thus, represents the typical N1-P2 complex that is elicited as a reaction to sound onset [57], [58]. The fact that for all stimuli the MUSIC component subspace (that represents 98% of the variance of the information ‘relevant’ for optimizing the EEG projection) contained a similar scalp pattern links our results to canonical onset-related ERP components and contributes to their neurophysiological interpretability

The scalp pattern of the present MUSIC component, in particular, resembles Schaefer et al.’s results where a fronto-central component occurred consistently in the decomposed grand average ERP response of participants who listened to fragments of naturalistic music. This group-level ERP had a time course that was correlated with the stimulus envelope with a time lag of 100 ms. Kaneshiro et al. [59] found a similarly distributed fronto-central component as most reliably occurring component in the EEG of listeners who were presented with naturalistic vocal music.

In contrast, the temporal dimension of the selected MUSIC components is much more variable across stimuli (see Fig 7). It is important to recognize that the time courses of MUSIC components differ from averaged ERPs (even though they are on the same time scale). They indicate the weighting of the corresponding scalp pattern over time and, thus, resemble a convolution model or FIR filter rather than an ERP time course. Thus, these time courses describe how information is integrated over time in the optimization process. Typically, such representations are viewed as an estimate of the impulse response of the auditory system [6]. Nonetheless, time lags with large weights in principle can be compared to latencies of canonical ERP components. In summary, our results suggest that in stimuli where a sizeable level of CACor is detected, the spatio-temporal patterns contain neural components that are neurophysiologically plausible and reminiscent of an N1-P2 complex. The variability of their temporal dimension suggests that the shape and latency of the N1-P2 response is influenced by stimulus properties, a finding which agrees with the sensitivity of the N1-P2 complex to several auditory features [2], [3].

### Between-stimulus comparison of CACor

The present approach represents an exploratory analysis; therefore, it is not known under which circumstances CACor can be detected. Onset-related brain responses may depend on a multitude of stimulus properties, most probably on the amplitude, tempo, attack, and regularity of the onsets. Technically, their detectability may depend on the number of samples (i.e., stimulus length) and on its stationarity. To gather experience, we applied the proposed method to a range of stimuli from different sound categories.

We utilized Ridge Regression with the audio power slope as a tool to investigate Cortico-Acoustic Correlation (CACor), the degree of synchronization of a listener’s EEG signal to the onset ‘landscapes’ of complex music and non-music stimuli. To assess a stimulus’ power to effect significant CACor we calculated CACor scores which are given by the number of single presentations with significant CACor (out of 27 presentations for all subjects in total). The CACor score ranking is led by the semi-musical repetitive Chord Sequence (CACor score 22/27), followed by the two romantic piano pieces by Chopin and Rachmaninov. These were followed by both Vivaldi stimuli, then by Bach and Williams. For the non-music stimuli significant CACor was present in none of the presentations. Remarkably, in the CACor score ranking (Fig 5A) stimuli from the same category, e.g., both pieces by Vivaldi or both romantic piano pieces are adjacent, suggesting that their acoustic/musical structure influences CACor in similar way. Descriptions of the stimuli with respect to average magnitudes of nine acoustic/musical features revealed that the CACor scores are significantly positively correlated with global measures of Sharpness (see Table 1), negatively with Spectral centroid. Sharpness can be described as a measuring how many ‘auditory edges’ a sound presents [30] and depends on the number and amplitude of peaks in the power slope. Spectral Centroid is related to the perception of brightness. Taken together, our results suggest that significant CACor is detected most reliably in stimuli with high onset density (containing a high number of note onsets) and with onsets that are characterized by a high contrast between baseline and peak sound intensity. Furthermore, lower frequencies in the stimulus seem to be more effective in ‘capturing’ the brain. Both, beat density and beat salience have been reported as strongly promoting the experience of ‘groove’ in songs [60]. An influential role of energy in low frequency bands in the induction of movement has been reported in [34]. In speech processing research Doelling et al. [30] pointed out a link between the Sharpness of ‘auditory edges’, strength of stimulus-tracking of cortical oscillations, and speech intelligibility

Even though the negative correlation with global Fluctuation entropy values and CACor score are not significant the relatively strong negative correlation indicates generally lower CACor scores for stimuli with complex rhythmic structure. This result points to an influence of rhythmic regularity on the synchronization of brain responses to the onset structure which is in line with onset-related ERP amplitudes for repetitive stimuli [61].

As a behavioral counterpart of CACor scores we examined Coordination scores, a measure of how strongly a stimulus effects similar trends in tension ratings in a group of subjects. CACor scores and Coordination Scores were significantly correlated. Note that Chord sequence is not included in this comparison, since no ratings were recorded for this stimulus. In contrast to the CACor scores, none of the global stimulus descriptions has a significant influence on the Coordination scores, even though correlation with Spectral centroid and Sharpness is still relatively high, but not significant. This means that while two acoustic factors have been identified that promote CACor, the consistent experience of tension in a group of listeners depends on more complex and variable configurations of musical parameters that, nevertheless, may encompass also those that have been linked to CACor in the present analysis. Thus, stimuli that leave a marked physiological reflection in the EEG, also lead to congruent perception of tension in different subjects. Tentatively, this might provide a link between (low-level) physiological reactions to sounds and the complex concept of musical tension.

#### Within-stimulus dynamics of CACor, music features and tension ratings

Considering the variable surface [62] of a naturalistic stimulus, it can be assumed that stimuli not only differ with respect to global measures of CACor, but also that the synchronization between brain signal and stimulus varies during the course of the stimulus. In order to learn how the dynamics of different acoustic/higher-level music features influence this synchronization we compared time-resolved CACor and the time courses of the nine acoustic/ higher-level music features for each stimulus. Our results amount to a small but distinct set of significant correlation coefficients. In particular, Sharpness, which was one of the main influences on CACor scores found in the between-stimulus comparison of CACor, and Spectral Entropy seem to modulate local within-stimulus CACor. This, again, points to an important role of ‘auditory edges’ and spectral properties in the cortical processing of note onsets. It demonstrates that even at this fine-grained time scale a direct relation of these properties to brain-stimulus synchronization can be detected.

Interestingly, Sharpness was the one feature that co-varied with tension ratings most consistently (see S2 Table), while Sound intensity, Spectral Entropy, and Fluctuation Entropy influenced tension ratings only in one stimulus each. This result adds new aspects to previous findings that identified loudness (which is closely related to Sound Intensity) as a main factor for the experience of tension [26] [25] [42] [63] [64]. In [21] harmonic and melodic structure were demonstrated to influence felt tension in addition, whereas their relative contribution depended on the specific stimulus. Our results show that tension is an even more multi-faceted phenomenon since Sharpness can be characterized as ‘acoustic’ (rather than related to higher-level musical structure) and rhythm-related.

A rather general notion of 'tension' has been associated with conflict, instability, or uncertainty [65]. Along these lines, a tentative explanation of the present influence of Sharpness on the experience of musical tension may be formulated: A change from a high level of Sharpness which is characterized by the presence of distinct salient ‘auditory edges’ to a passage where musical events are less clearly defined can be thought to produce uncertainty in the listener and may lead to an increase in experienced tension.

From the perspective of tension as a possible link between predictive processes and emotion [65] [66] our findings may also be further interpreted as reflecting different degrees of predictability of future events: Salient auditory edges (that in our stimuli often go together with clear rhythm) promote the formation of rhythmic and metric predictions. However, the interplay between expectation, fulfilment or surprise that is thought to lead to emotional responses to music is complex and not yet fully understood [21]. Still, this theory may provide a tentative explanation how a stimulus property that can be considered as a ‘merely’ acoustic one contributes to an emotion-related aspect of experiencing music.

The set of nine stimuli in this exploratory analysis contained a wide range of different sounds. Of these, two full length romantic piano pieces represented the stimuli for those influences of stimulus structure, both, on brain-stimulus synchronization and tension ratings was detected most consistently. Acoustically, this can be related to the fact that the piano, as a struck string instrument, has a characteristic attack ‘thump’ [67] and therefore is likely to provide salient onsets that are reflected well in the brain response. Remarkably, the two piano pieces (within our set of stimuli) represent the most complex musical surfaces as (typically for this period) they contain long, lyrical melodies, wide skips, chromaticism, strong contrasts, expressive performance and interpretive freedom (rubato). These highly complex pieces reveal a relation between stimulus structure and physiological measures and behavioral measures most clearly. To a lesser extent, this relation is visible in two movements of a baroque violin concerto, that feature a rhythmic regular pulse and a contrast between ‘solo’ passages of the solo violin and ‘tutti’ passages of the orchestra. Taken together, this suggests that structural variance/richness and strong contrasts are aspects of music that give rise to a clear physiological and behavioral reflection of structural elements of music, a finding that represents a strong argument for experimental paradigms using full-length naturalistic music stimuli.

### Limitations

The present results are a proof-of-concept that multivariate methods of EEG analysis can achieve considerable advances in extracting onset-related brain responses from the ongoing EEG, enabling more naturalistic experimental paradigms. Notwithstanding, several issues call for further exploration. Our results have demonstrated that complex stimuli with different characteristics vary with respect to the consistency of the occurrence of CACor in a group of subjects. The present analysis has identified acoustic properties that are related to these differences. However, the variance of CACor between presentations within subjects that occurred in artifact-cleaned data has not been explained yet. Onset-related ERPs are known to be influenced by a range of subject variables, some of them being situation-dependent, such as attentional state vigilance or familiarity ([68], [69], [56]). Furthermore, CACor, as a single-trial technique, is also affected by the distribution of noise in the EEG: An absence of significant CACor in a single presentation may have many reasons and therefore cannot be uniquely interpreted. Yet, the relative frequency of occurrence of significant CACor in a collection of presentations (e.g., by deriving CACor scores) is a promising way to estimate the capability of a musical composition to forcefully drive its processing in a listeners' brain.

The present analysis revealed that EEG signals may significantly reflect the onset structure of music, and, that, if this is the case, also the dynamics of tension ratings are consistent for a group of subjects. At a more detailed level (within stimuli), however, we found only weak relations between CACor and reported tension. This means that the onset structure of music can ‘drive’ cortical activity, but that it is not clear whether and how this impacts on conscious experience of musical tension. Beyond the concept perceived tension, further measures related to experience of music may be required for investigating this aspect in detail. Behaviorally reported qualities describing listening experience, such as immersion or involvement, could be evaluated in future experiments. In addition, it would be interesting to probe whether stimuli effecting significant CACor also synchronize other physiological parameters, such as heart rate and respiration.

The group of subjects who took part in this exploratory study was relatively small and heterogeneous with respect to several factors. However, in the analysis we focus on detecting those parts of the brain signal that directly relate to acoustic properties and can be assumed to represent basic sensory processing. These processes should occur with a certain consistency in any subject. Furthermore, our main results relate to differences between stimuli. Here, the heterogeneity of the group, in principle, could weaken our results. However, we find clear differences between stimuli, at both, the EEG level and the behavioral level. Our results show that for this kind of analysis a heterogeneous group of subjects is not problematic, but, on the contrary, even reveales the robustness of the observed effect.

### Outlook

In principle, differences in single-presentation CACor may reflect a range of variables, including stimulus properties, various user characteristics, such as age, training, familiarity, preferences, but also situational factors, such as attention. Thus, within one stimulus, the proposed method could be a promising instrument to investigate the influence of such factors on sensory processing of music by comparing the CACor scores of different populations, such as musicians versus non-musicians, different age groups or connoisseurs of a specific genre versus subjects with a ‘neutral’ attitude. Critically, the chance of presenting and analysing continuous music pieces in a single-trial setting represents a qualitative change in experimental design: It opens up new avenues for research into aspects of experiencing music that cannot possibly accessed with short, simplified and repeated stimuli.

#### Conclusion

The present results demonstrate that sequences of note onsets which constitute original complex music can be reconstructed from the listener’s EEG using spatio-temporal regression-derived filters, leading to a significant Cortico-Acoustic Correlation at the level of single subjects and single presentations. The characteristics of the extracted brain signatures represent a link to canonical onset-related ERPs, such as the N1-P2 complex, that can be extracted by averaging techniques. Stimuli that consistently led to significant Cortico-Acoustic Correlation had high level of Sharpness, were rhythmically simple, or were dominated by low-frequency content. This points to an important role of the presence of a simple salient beat and percussive elements in physiological reactions to music. The same stimuli produced strongly coordinated ratings in group of 14 listeners, a finding that provides a tentative link from Cortico-Acoustic Correlation to conscious experience of music. At a temporally more resolved level this relationship between CACor and stimulus properties was confirmed, while tension ratings seemed to reflect the same properties differently. These results show that the proposed approach enables to investigate the processing of brain responses to tone onsets in complex sounds with enhanced sensitivity and, thus, may represent a valuable tool in a range of applications where naturalistic listening scenarios are of interest.

## Supporting Information

S1 Fig Example of decomposition of regression patterns into MUSIC components. Top: Spatio-temporal pattern derived directly from regression filters for the Chord sequence and subject S2. Bottom: Decomposition of the spatio-temporal pattern into MUSIC components (MUSIC algorithm) with a spatial and a temporal dimension each. The values on the right side indicate the percentage of variance the underlying PCA component covers.
(TIF)

S2 Fig Overview on data types and levels of analysis. At the level of single presentations (2^nd^ column) one CACor coefficient is calculated for each stimulus presentation. At the Between-Stimulus level (3^rd^ column) global values for each stimulus (tension ratings: Coordination score, EEG data: CACor score, audio analysis: global music features) are aggregated into a profile for the set of nine stimuli. The correlation between these profiles is calculated. At the Within-Stimulus level average tension ratings, group-level time-resolved CACor and time courses of the extracted music features are correlated for each stimulus. The paragraphs in the text that refer to the corresponding steps of analysis/results are given in grey.
(TIF)

S3 Fig Music feature profiles for nine acoustic/higher-level music features. The bars indicate the average magnitude of a music feature for the set of nine stimuli. This illustrates differences in global stimulus characteristics.
(TIF)

S4 Fig Scalp topographies of selected MUSIC components for all subjects and stimuli. (TIF)

S1 Table List of stimuli. (DOCX)

S2 Table (a) Partial correlation of time-resolved CACor of Grand Average with nine music features. Pink shading indicates significant positive partial correlation, blue shading indicates significant negative partial correlation (alpha = 0.05). (b) Partial correlation of mean tension ratings with nine music features. Pink shading indicates significant positive partial correlation, blue shading indicates significant negative partial correlation (alpha = 0.05). (TIF)
