# Supplementary figures and images for: Multi-Variate EEG Analysis as a Novel Tool to Examine Brain Responses to Naturalistic Music Stimuli
Source: PLoS One. 2015 Oct 28;10(10):e0141281. doi: 10.1371/journal.pone.0141281 (PMC4624980; doi:10.1371/journal.pone.0141281)

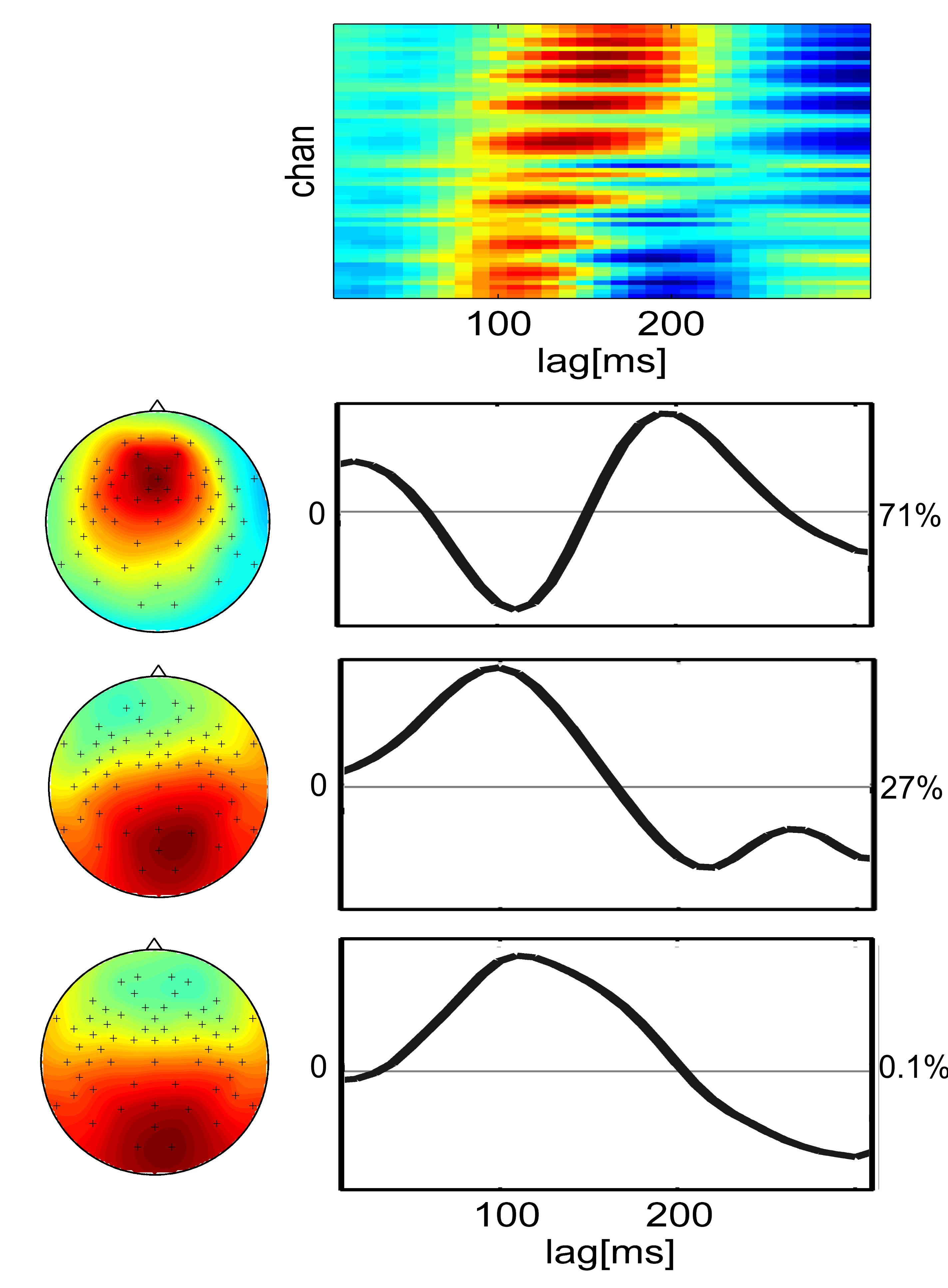

Supplement: S1 Fig — Top: Spatio-temporal pattern derived directly from regression filters for the Chord sequence and subject S2. Bottom: Decomposition of the spatio-temporal pattern into MUSIC components (MUSIC algorithm) with a spatial and a temporal dimension each. The values on the right side indicate the percentage of variance the underlying PCA component covers. (TIF) [file pone.0141281.s001.tif]

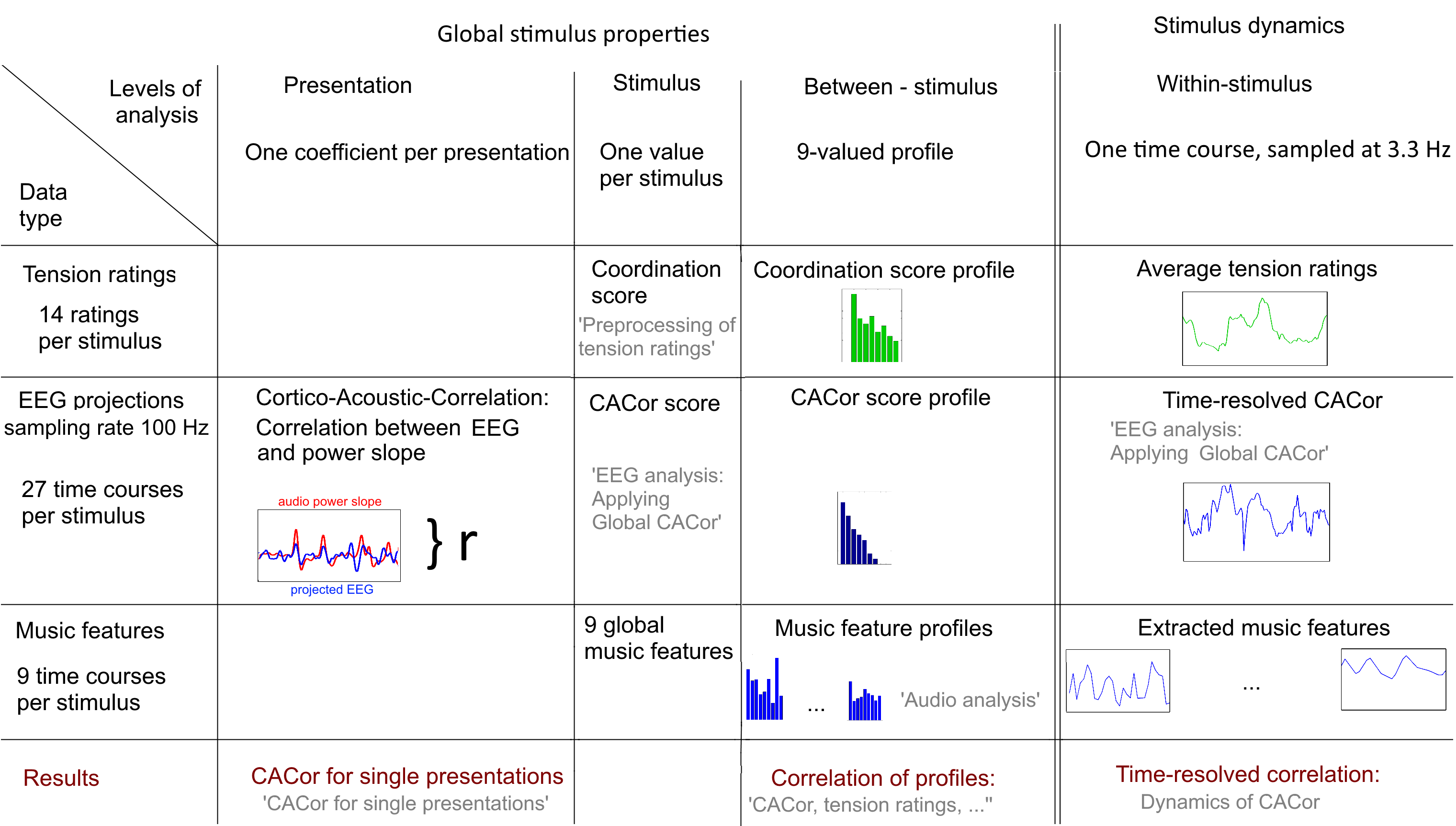

Supplement: S2 Fig — At the level of single presentations (2nd column) one CACor coefficient is calculated for each stimulus presentation. At the Between-Stimulus level (3rd column) global values for each stimulus (tension ratings: Coordination score, EEG data: CACor score, audio analysis: global music features) are aggregated into a profile for the set of nine stimuli. The correlation between these profiles is calculated. At the Within-Stimulus level average tension ratings, group-level time-resolved CACor and time courses of the extracted music features are correlated for each stimulus. The paragraphs in the text that refer to the corresponding steps of analysis/results are given in grey. (TIF) [file pone.0141281.s002.tif]

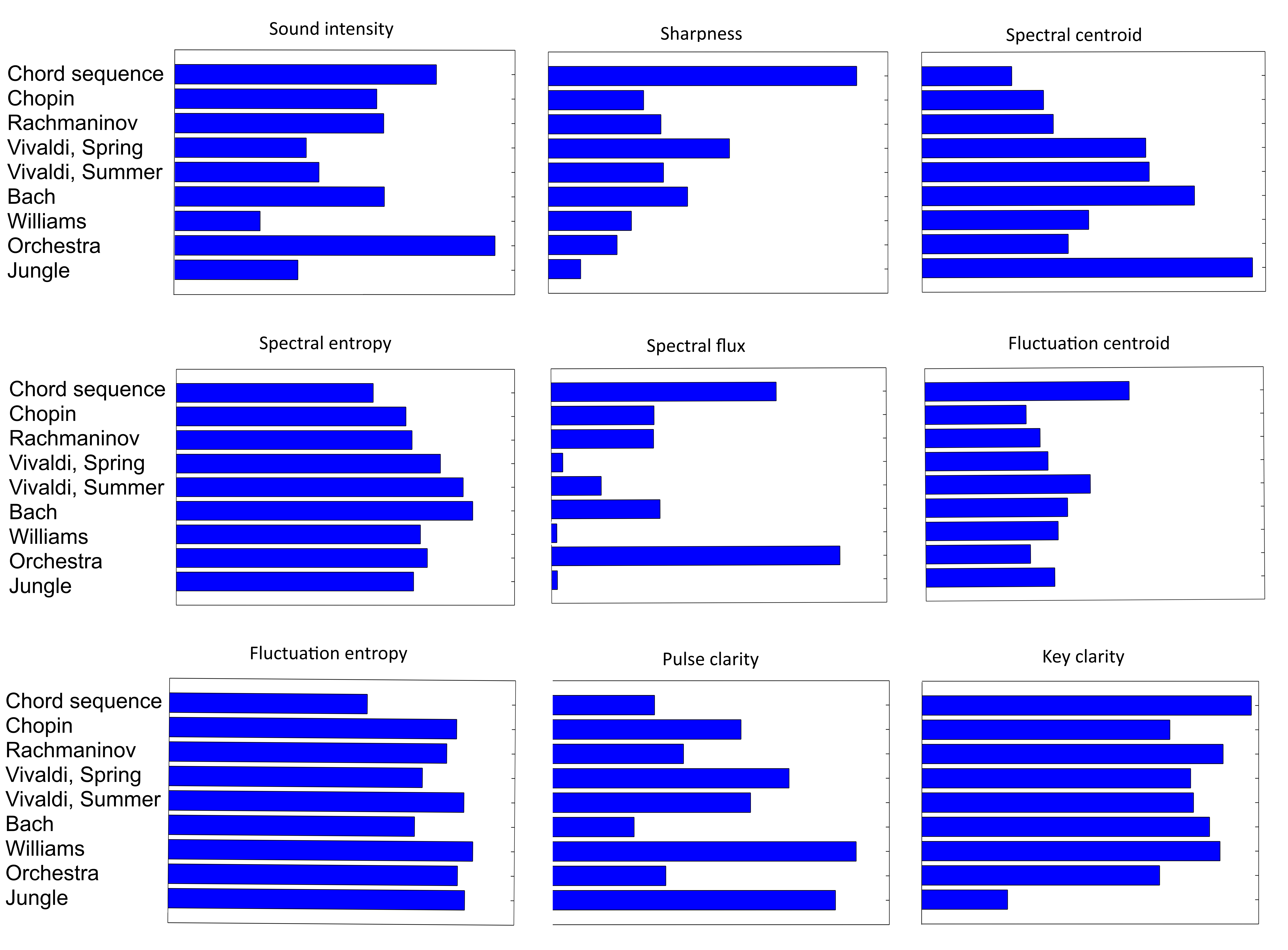

Supplement: S3 Fig — The bars indicate the average magnitude of a music feature for the set of nine stimuli. This illustrates differences in global stimulus characteristics. (TIF) [file pone.0141281.s003.tif]

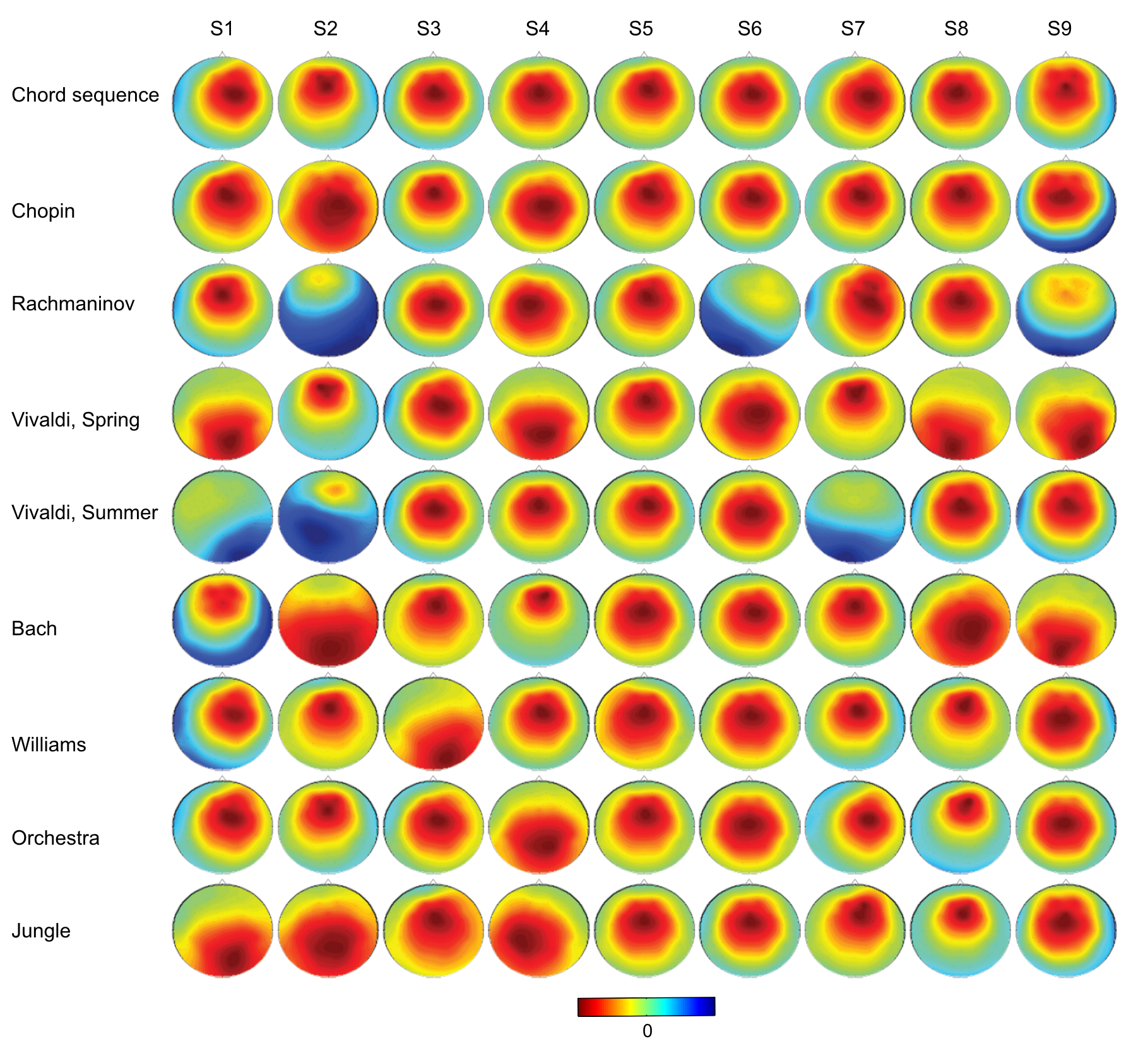

Supplement: S4 Fig — (TIF) [file pone.0141281.s004.tif]

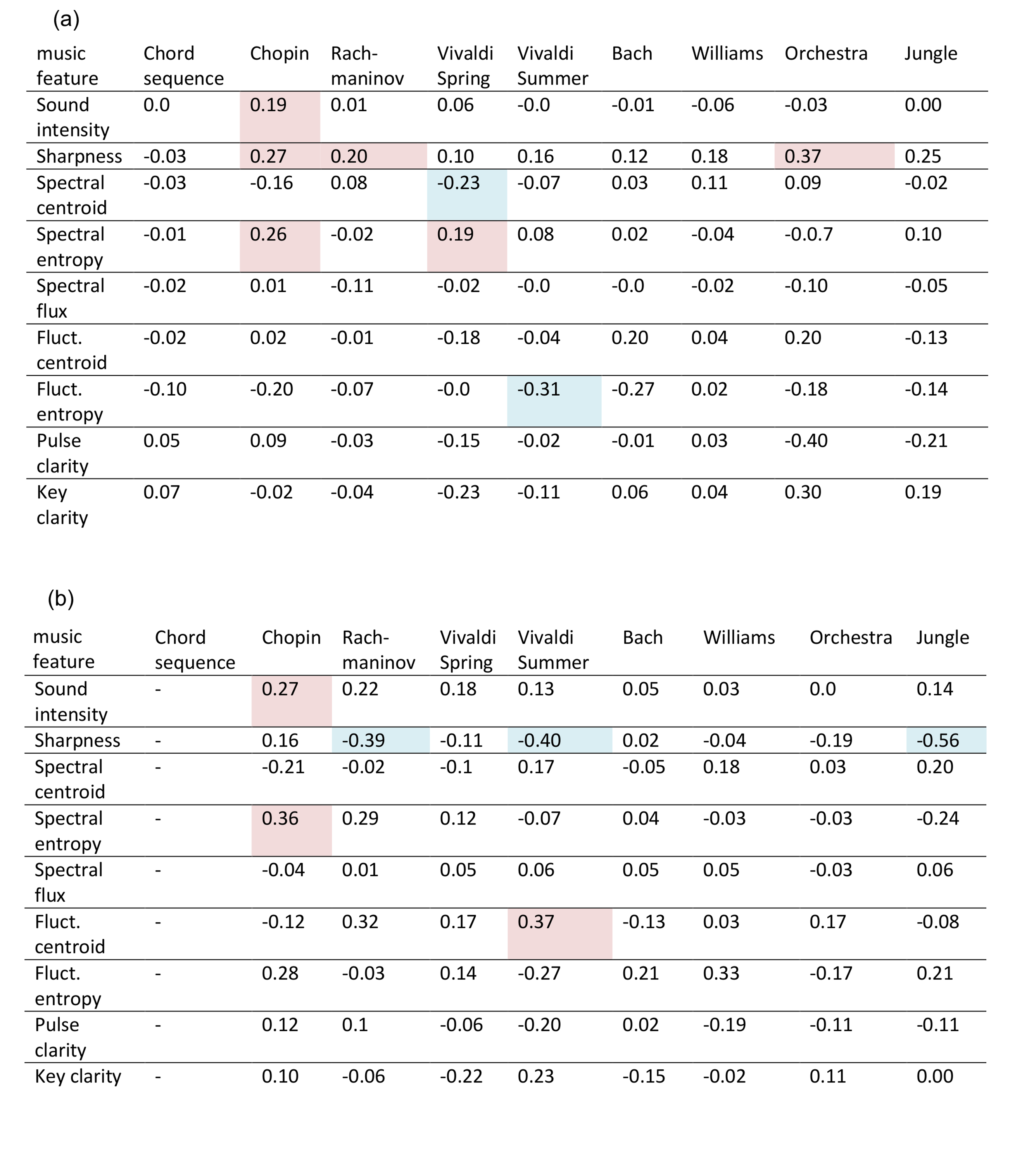

Supplement: S2 Table — (TIF) [file pone.0141281.s006.tif]
