# Supplementary material for: Multi-Variate EEG Analysis as a Novel Tool to Examine Brain Responses to Naturalistic Music Stimuli
Source: PLoS One. 2015 Oct 28;10(10):e0141281. doi: 10.1371/journal.pone.0141281 (PMC4624980; doi:10.1371/journal.pone.0141281)
Supplement: S1 Table — (DOCX) [file pone.0141281.s005.docx]

| name | composer | title | duration | Recording/source | description |
| --- | --- | --- | --- | --- | --- |
| Bach | J.S.Bach | Badinerie | 1’ 20’’ | T. Koopman,  Amsterdam Baroque Orchestra,  Image Ent.,1995 | From Orchestral Suite No. 2 in B minor, BWV 1067 for Flute, strings and basso continuo |
| Vivaldi, Spring | 1. Vivaldi | The Four Seasons, op. 8, Spring | 3’ 12’’ | P. Schoeman,  London Philharmonic Orchestra  X5 Music Group,  2010 | Concerto for Violin and Orchestra |
| Vivaldi, Summer | 1. Vivaldi | The Four Seasons, op. 8, Summer | 2’ 49’’ | A.Loveday,  Academy of St. Martin-in-the-fields, Decca, 1969 | Concerto for Violin and Orchestra |
| Chopin | F. Chopin, | Etude op. 12, No. 10 | 2’ 39’’ | V. Horowitz, Sony 1997 | Piano solo |
| Rachmaninov | S.Rachmaninov | Prelude op. 32, No. 5 | 3’ 44’’ | E. Gilels, live recording,  Moscow, 1967 | Piano solo |
| Williams | J. Williams | Theme of Schindler’s List for violin and orchestra | 3’ 31’’ | I. Perlman,  MCA Records, 1994 | Violin and Orchestra |
| Chord sequence |  |  | 3’ 8’’ | generated in Matlab | Sequence of major triad chord on all tones of the chromatic scale. After 7-11 repetitions of a chord change to a new chord in random manner |
| Orchestra |  | Sound of symphony orchestra tuning in/playing | 1’ 04’’ | http://www.youtube.com/watch?v=IslDWrmOieE | Sound of an orchestra playing/tuning in before a performance |
